# Synthesis, Structure and Biological Activity of Indole–Imidazole Complexes with ZnCl_2_: Can Coordination Enhance the Functionality of Bioactive Ligands?

**DOI:** 10.3390/molecules28104132

**Published:** 2023-05-16

**Authors:** Karolina Babijczuk, Beata Warżajtis, Justyna Starzyk, Lucyna Mrówczyńska, Beata Jasiewicz, Urszula Rychlewska

**Affiliations:** 1 Department of Bioactive Products, Faculty of Chemistry, Adam Mickiewicz University, Uniwersytetu Poznańskiego 8, 61-614 Poznań, Poland; karolinababijczukk@gmail.com; 2 Department of Crystallography, Faculty of Chemistry, Adam Mickiewicz University, Uniwersytetu Poznańskiego 8, 61-614 Poznań, Poland; beataw@amu.edu.pl; 3 Department of Soil Science and Microbiology, Faculty of Agronomy, Horticulture, and Bioengineering, University of Life Science, Szydłowska 50, 60-656 Poznań, Poland; justyna.starzyk@up.poznan.pl; 4 Department of Cell Biology, Faculty of Biology, Adam Mickiewicz University, Uniwersytetu Poznańskiego 6, 61-614 Poznań, Poland; lumro@amu.edu.pl

**Keywords:** indole derivatives, Zn(II) complexes, human erythrocytes, oxidative hemolysis, antibacterial activity, fungicidal properties

## Abstract

The ability of the indole–imidazole hybrid ligands to coordinate with the Zn(II) ion and the resulting structures of this new class of coordination compounds were analyzed in order to determine their structural properties and biological functionalities. For this purpose, six novel Zn(II) complexes, [Zn(InIm)_2_Cl_2_] (**1**), [Zn(InMeIm)_2_Cl_2_] (**2**), [Zn(IniPrIm)_2_Cl_2_] (**3**), [Zn(InEtMeIm)_2_Cl_2_] (**4**), [Zn(InPhIm)_2_Cl_2_] (**5**) and [Zn_2_(InBzIm)_2_Cl_2_] (**6**) (where InIm is 3-((1H-imidazol-1-yl)methyl)-1H-indole), were synthesized by the reactions of ZnCl_2_ and the corresponding ligand in a 1:2 molar ratio in methanol solvent at an ambient temperature. The structural and spectral characterization of these complexes was performed using NMR, FT–IR and ESI–MS spectrometry and elemental analysis, and the crystal structures of **1**–**5** were determined using single-crystal X-ray diffraction. Complexes **1**–**5** form polar supramolecular aggregates by utilizing, for this purpose, the N-H(indole)∙∙∙Cl(chloride) intermolecular hydrogen bonds. The assemblies thus formed differ depending on the distinctive molecular shape, which can be either compact or extended. All complexes were screened for their hemolytic, cytoprotective, antifungal, and antibacterial activities. The results show that the cytoprotective activity of the indole/imidazole ligand significantly increases upon its complexation with ZnCl_2_ up to a value comparable with the standard antioxidant Trolox, while the response of its substituted analogues is diverse and less pronounced.

## 1. Introduction

Indole is one of the most common heterocyclic compounds in nature. Indole occurs in many biologically important natural products, e.g., in the neurotransmitter serotonin, the plant growth hormone auxin, or in alkaloids, such as vinblastine, reserpine, or gramine [1,2,3,4]. In addition to its important role in cell signaling, this molecule is a versatile building block for the synthesis of new bioactive and pharmaceutical compounds [5,6,7].

Many compounds containing indole moiety have been reported to exhibit anti-inflammatory, antibacterial, antiviral, and antioxidant activities [5,6,7,8,9,10,11]. 

Imidazole and its derivatives are one of the most important and widely used heterocycles in medicinal chemistry, natural products, and synthetic chemistry. For the most recent review on the subject see [12]. Imidazole is the basic core of natural products such as histidine, purine, histamine and DNA-based structures [13]. Imidazole derivatives possess an extensive spectrum of biological activities, such as antibacterial, anticancer, antitubercular, antifungal, analgesic and anti-HIV.

Several imidazole derivatives have been widely used in the clinic to treat a wide range of illnesses, suggesting their enormous potential for further research [14]. 

Recently, indole–imidazole hybrids have attracted more attention. This type of conjugate shows antioxidant, antimicrobial, and anticancer properties [15,16,17,18,19,20]. As drugs, they have been used as α-glucosidase inhibitors [21] and selective 5-HT7R agonists [22].

Bioactive metal complexes can play a valuable role in drug design. The diverse geometry of these complexes and their ability to exchange ligands, combined with the therapeutic properties of metal ions and ligands, can lead to the synthesis of many new therapeutics with properties that are not found in the ligands alone. Zn complexes are known for their antimicrobial [23,24,25,26,27,28], anticancer [29,30], and antioxidant properties [24,30,31,32,33,34,35]. The therapeutic effects of drug–metal complexes are not only often comparable to those of conventional drugs, but they are also more active, selective, bioavailable, and have fewer side effects [36]. One example is the indole derivative indomethacin. This non-steroidal anti-inflammatory drug can cause damage to the digestive tract, leading to ulcers. Complexing indomethacin with zinc can effectively counteract this side effect without compromising the therapeutic effect [37,38,39]. In addition, zinc itself affects the functioning of the body, e.g., decreasing blood pressure and heart rate [40]. Zinc ions are located in the active center of many enzymes, enhancing the catalytic activity of the enzyme or stabilizing the structure [40]. For example, zinc-binding histidine sequences in calprotectin inhibit microbial growth by competing for zinc [41]. In addition, the stability of Zn(II) and the lack of redox activity under biological conditions make the zinc ion an ideal metal for many reactions. 

Nowadays, compounds with antioxidant capacities are of great interest, especially for preventing the adverse effects caused by free radicals in cells. The antioxidant activity of metal complexes may be due to the biological activity of the metal and the ligands bound to it [40,42]. The ability of zinc to inhibit oxidative processes is well known [43]. Although zinc does not directly interact with the oxidant, it may indirectly reduce its effect by protecting cells from the oxidative damage and stabilizing the molecular structure of the cell membrane [44]. Our previous work has shown that indole derivatives with a substituent at the C-3 position protect human erythrocytes against oxidative hemolysis in vitro [8,16,45,46]. In addition, indole–imidazole compounds exhibit excellent iron complexing properties [16]. 

The metal-binding capacity of antioxidants can reduce the concentration of transition metals, such as Cu and Fe, that catalyze Fenton or Haber–Weiss reactions, forming reactive oxygen species (ROS) such as hydroxyl radicals or superoxide anions.

For all of these reasons, we considered it worthwhile to validate the synergic integration of 3-substituted indole (an analogue of the natural alkaloid gramine) and imidazole derivatives, and to compare the biological activity of the hybrid ligands thus obtained with the biological activity of their Zn(II) complexes.

We have synthesized and characterized six new complexes of zinc with chloride and indole–imidazole hybrids as ligands. The chloride was chosen as the counterion because of its presence in the extracellular and intracellular environment.

The complexes were characterized via single-crystal X-ray analysis and various spectroscopic methods. The cytoprotective activity of the derivatives was evaluated in vitro by determining the inhibition of oxidative-hemolysis, which is induced by free radicals generated by 2,2′-azobis(2-amidinopropane hydrochloride) (AAPH). Human erythrocytes (red blood cells, RBCs), due to the lack of any intracellular membrane systems, are widely used as model cells to assess the protective activity of the cell membrane-incorporating compounds under oxidative stress conditions [8,16,45,46]. In addition, the complexes were screened for antibacterial and antifungal properties. The results gained from this study were compared with those obtained recently for the uncomplexed ligands. The effect of substitution in the imidazole ring was also investigated. 

## 2. Results and Discussion

### 2.1. Synthesis and Spectroscopic Characterization of ZnCl_2_ Complexes with Indole–Imidazole Ligands

**Scheme 1:**
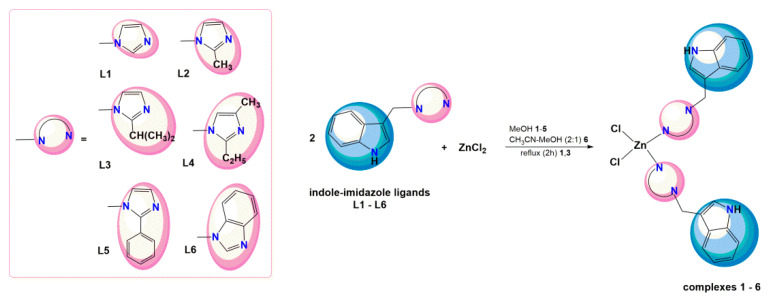
Synthetic route of ZnCl_2_ complexes with indole–imidazole ligands.

The indole–imidazole ligands, including (InIm) (**L1**), (InMeIm) (**L2**), (IniPrIm) (**L3**), (InEtMeIm) (**L4**), (InPhIm) (**L5**) and (InBzIm) (**L6**), where InIm is 3-((1H-imidazol-1-yl)methyl)-1H-indole (see Scheme 1), were synthesized from gramine and substituted imidazoles according to our previous report [16]. The reactions of the obtained ligands with anhydrous ZnCl_2_ afforded complexes **1**–**6** in good yields (Scheme 1). The complexes were characterized using spectroscopic methods (NMR, FT–IR), ESI–MS spectrometry, melting point, and elemental analysis. 

All complexes were crystallized in a 2:1 ratio (ligand: ZnCl_2_). The NMR spectra (in DMSO-*d*_6_) of the synthesized complexes show one set of signals corresponding to the coordinated ligand, indicating that the ligands are equivalent. Furthermore, their similarity to the ligand spectra suggests that the indole–imidazole compounds in the resulting complexes remain structurally unchanged in solution.

The ^13^C NMR spectra of the complexes showed resonances due to the indole, imidazole, and benzimidazole carbon atoms in the regions of 109–136, 118–152, and 108–144 ppm, respectively. The signals from the phenyl carbon atoms (complex **5**) range from 126 to 130 ppm, while the signals from the methylene C(10) carbon atom appear near 40 ppm. The carbon atoms of the alkyl substituents appear in the range of 12–25 ppm. In the ^1^H NMR spectra of complexes **2**, **3**, and **4**, the signals from methyl, ethyl, and isopropyl groups were assigned to 1.99–2.45, 1.09–2.90, and 1.20–3.41 ppm, respectively. The signals of indole NH protons were found near 11 ppm. Indole aromatic ring protons were observed at 7.57–6.38 ppm, while those of imidazole (**2**–**5**) and benzimidazole (**6**) were detected at 6.84–8.19 ppm and 7.21–8.78 ppm, respectively. C(10)H_2_ protons appeared at 5.22–5.73 ppm. 

The FT–IR spectra of all complexes show a broad band at 3600–3400 cm^−1^ (N-H indole) and a signal at 650–540 cm^−1^, characteristic of the indole ring. Due to the presence of hydrogen bonds in the complexes, the band produced by νN-H (indole ring) is more intense and is shifted to lower wavenumber values than those in the spectra of the ligands [16]. 

In the ESI–MS spectra of all compounds, the signal of [Zn(L)_2_Cl]^+^ was present. The molecular composition of complexes **1**–**6** was also confirmed via elemental analysis. The NMR, FT–IR, and ESI–MS spectra of all investigated complexes are provided in the Supplementary Materials (Figures S1–S18).

### 2.2. X-ray Analysis

**Figure 1:**
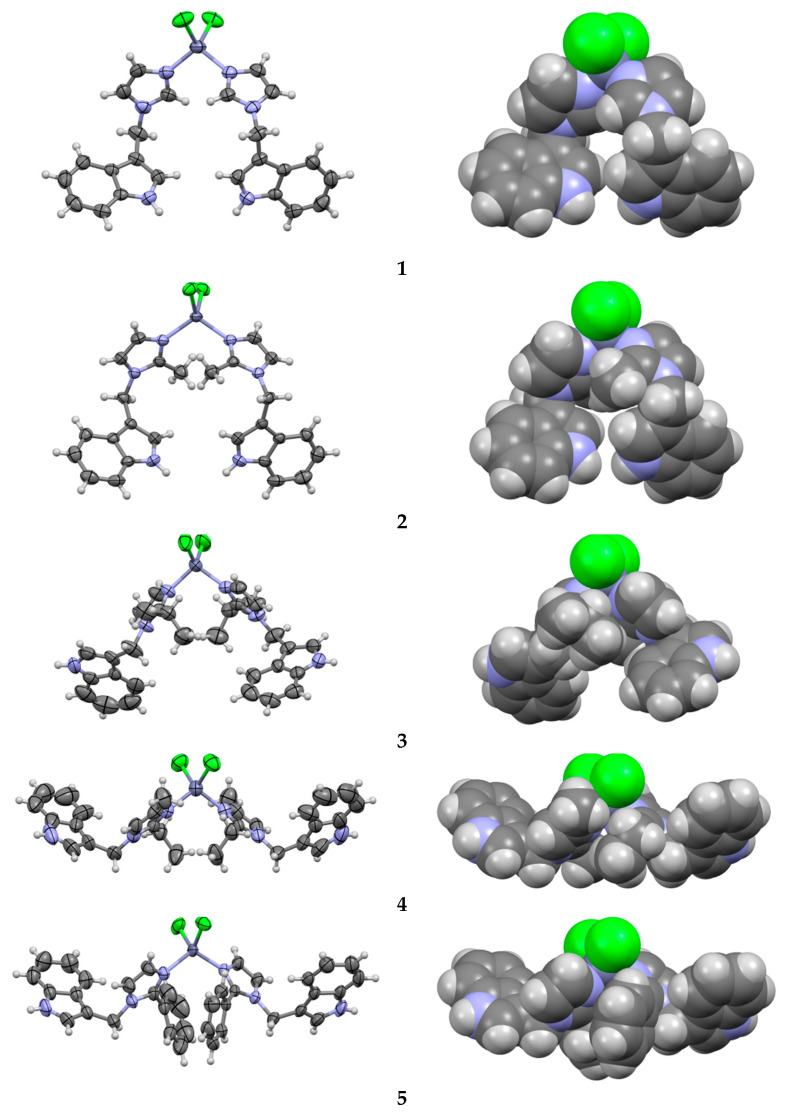
Molecular conformation, as present in the crystals of compounds **1**, **2**, **3**, **4** and **5** in two representations, i.e., with thermal ellipsoids, drawn at the 30% probability level and in a space-filling mode.

Single crystals suitable for X-ray diffraction were obtained for five complexes (**1**, **2**, **3**, **4** and **5**). The crystals were grown from MeOH. The structures of the molecules, as seen in crystals, are shown in Figure 1.

The Zn(II) ion is tetrahedrally surrounded by two imidazole nitrogen atoms of two hybrid indole–imidazole ligands and two chloride ions. The bond lengths and angles within the coordination sphere are listed in Table S1. It might be worth noting that the spread of the values of the valence angles around Zn(II) is considerably larger in molecules **3** and **4**, which contain highly substituted imidazole rings compared to **1**, **2** and **5**. The narrowest (102.68(12)°) and widest (115.13(6)°) values of the valence angles appear in molecule **3**, and concern the N–Zn–N and Cl–Zn–Cl angles, respectively. This is likely a consequence of the steric requirements caused by the isopropyl substitution within the imidazole part of the ligand. Hence, complexes **1**–**5** can be described as having a distorted tetrahedral environment. Formally, the highest symmetry of the investigated complexes is *C*_2*v*._ In crystals, the molecules either utilize *C*_2_ (**1**, **2**, **4**) or *C*_1_ symmetry (**3** and **5**). Of the two asymmetric molecules **3** and **5**, the latter approximates to the formal *C*_2_ symmetry, while the former is highly asymmetric. The various conformations of the hybrid indole–imidazole ligands have been characterized by us previously [16] using a set of the ring-twist angles, i.e., the signed torsion angles φ_1_ and φ_2_ measured along the C–C and C–N bonds formed by the methylene bridge. In this paper, we have used the same descriptors to characterize the conformations of the ligands coordinated to Zn(II) and to assess to what extent the molecular conformation of the ligand changes upon coordination to the metal atom. These descriptors are listed in Table 1, which additionally provides the values of the torsion angle φ_3_ describing the mutual disposition of the indole–imidazole ligands in their complexes with ZnCl_2_.

In complexes **1**–**5**, the absolute values of φ_1_ vary from 68.1 to 97.5°, with a mean value of 80.5°, while those of φ_2_ cover a much wider range of 14.5 to 87.2°, with a mean of 49.4°. The highest absolute values of φ_2_ appear in the complexes that maintain the formal *C*_2_ molecular symmetry in their crystals, and the lowest appear in a highly asymmetrical complex **3**, which might result from the symmetry requirements. 

By having access to the structures of the uncoordinated ligands [16], we were able to monitor in two cases, i.e., **3** and **5**, the changes in the ligand structure that take place upon its coordination to the metal ion. In **5**, the alterations are negligible: the φ_1_ and φ_2_ torsion angles stay paired in sign and their absolute values are comparable to those of the uncoordinated ligand. In contrast, the complexation of a ligand with a iPr substituent (**3**) results in an increase in the absolute value of φ_1_ and accompanies a decrease in φ_2_. In one of the coordinated ligands, the two angles are no longer paired in sign, as they are in a free ligand. All these changes illustrate the marked asymmetrization of molecule **3**. 

As far as the spatial structure of the complex molecules is concerned, one can clearly distinguish the *C*_2_ symmetric molecules that have a compact shape (**1** and **2**), a molecule that has a relatively compact but highly asymmetric shape (**3**), and molecules that have an either precisely (**4**) or roughly (**5**) symmetrical shape with an extended structure (Figure 1). In molecules belonging to the first group, the indole fragments are directed inwards into the molecule, so the two H atoms from the pyrrole N-H groups are only 3.65 Å apart. The maximum distance between the peripheral indole H-atoms, which can be a measure of the molecular wideness, is about 14 Å. In contrast, molecule **3**, which has a noticeably asymmetrical shape, maintains the two indole fragments oriented inwards; however, their N-H groups are put aside and directed outwards. The intramolecular distance between their H-atoms, which approximates to 15 Å, can also be a measure of the molecular width. The molecules with a fully extended conformation (**4** and **5**) resemble a two-bladed propeller in their shape. They have their indole rings expelled to the periphery, with a maximum distance of approximately 19 Å between their peripheral H-atoms; meanwhile, there is a distance of approximately 17 Å between the H-atoms and from the two N-H groups. The spatial structure of the complex molecules determines the way in which they associate with into supramolecular aggregates, either in crystals or other condensed matter, or possibly interact with the receptor molecules. 

Packing in crystals is mostly governed by the intermolecular N-H(indole)···Cl(chloride) hydrogen bonds. Hydrogen bond parameters are listed in Table 2. 

**Figure 2:**
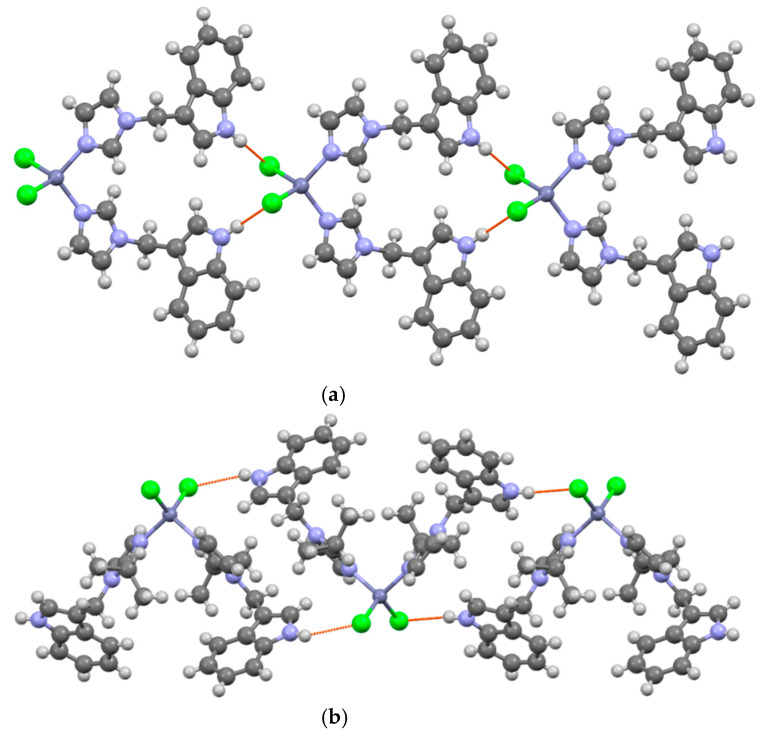

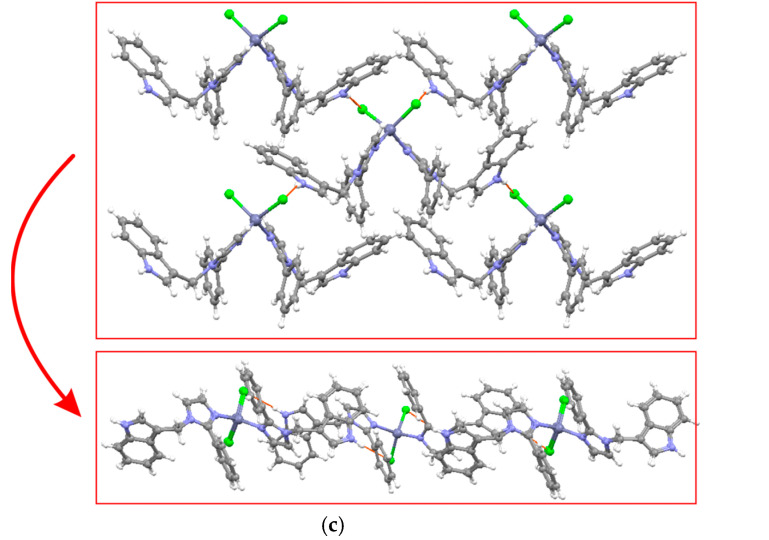
Illustration of the three different types of supramolecular aggregates formed in the crystals of **1**–**5** as a function of the molecular shape. (**a**) hydrogen-bonded polar chains, (**b**) tapes formed around the symmetry centers, (**c**) polar layered aggregates viewed perpendicularly and along the layer. The red arrow indicates the two views.

The molecules with a compact shape (**1** and **2**) formed two N-H···Cl hydrogen bonds to the same molecule, which were related by a single translation. As a result, the hydrogen-bonded molecules arranged into chains that extended along the polar *c*-direction, with chloride ligands pointing to its negative end (Figure 2a). Molecule **3**, with a less compact but not yet fully extended shape, formed hydrogen-bonded tapes, which were built from two different sets of centrosymmetric dimers. The dimers were formed by bonding each molecule to two neighboring molecules by means of four hydrogen bonds (Figure 2b). As listed in Table 2, the H-bond parameters for each dimer were noticeably dissimilar, which is in line with the marked asymmetry of molecule **3**. (Table 1). This type of packing is not very efficient, as indicated by the presence of small voids of the volume of 34.2 Å^3^ around the lattice points (as calculated with the use of Mercury [47] software using a contact surface option and a probe radius of 1.2 Å). In contrast, each of the complex molecules with an extended shape and indole N-H groups expelled outwards (**4** and **5**) formed N-H···Cl hydrogen bonds with four neighboring molecules, thus building the hydrogen-bonded layers (Figure 2c). The layers are polar, i.e., all of the ZnCl_2_ fragments are oriented in the same *b*-direction. Since the crystals are centrosymmetric, the neighboring layers have this fragment reversely oriented.

### 2.3. Biological Activity 

#### 2.3.1. Hemolytic Activity

Human RBCs (Red Blood Cells) are the most abundant cells in human blood and are widely used as an efficient cell model to evaluate the cytotoxic effects of natural and synthetic compounds. The hemolytic assay is mandatory in order to assess the hemobiocompatability of compounds for potential biomedical applications. Compounds that induce hemolysis by more than 5% at a given concentration are defined as non-hemocompatible compounds [8,16,45,46]. 

Compared to the starting ligands, the zinc chloride complexation meaningfully altered the hemolytic activity of the resulting complexes in only two cases (**5** and **6**). Changes in the hemolytic activity depend on the nature of the substituent present in the imidazole ring of the ligand and on the structure of the resulting complex. For complexes **1**–**4**, which contain an unsubstituted (**1**) or electron-donor-substituted (**2**–**4**) imidazole ring in the ligand structure, the hemolytic activity decreased slightly (complexes **1**–**2**) or did not change (complexes **3**–**4**); this is despite the significant changes in the structure of ligand **L3** that took place upon its complexation with Zn(II). 

Complexes **1**–**4** at a concentration of 0.1 mg/mL did not induce hemolysis at a rate higher than 5%; therefore, they are hemocompatible and can be used for the further evaluation of their biological activity. The hemolytic activity of complex **5**, which has an electron-withdrawing phenyl substituent at the imidazole ring, increased relative to the ligand activity (from 5.46% ± 1.09 to 9.14% ± 0.48, respectively), although the structural changes that took place within the ligand upon complexation with metal were small. The largest (almost twofold) decrease in hemolytic activity was observed for complex **6** with the benzimidazole substituent (from 23.38% ± 2.60 to 12.96% ± 0.90, respectively). 

In conclusion, complexes **1**–**4**, with or without electron-donating substituents in the imidazole ring, are hemobiocompatible, as their hemolytic activity at the concentration used (0.1 mg/mL) is below 5%, and they are good candidates for further evaluation. Complexes **5** and **6**, containing electron-withdrawing groups, were found to be cytotoxic (hemolytic activity >5%) and possess cell membrane-disrupting activity, meaning that they are not hemobiocompatible compounds.

#### 2.3.2. Cytoprotective Activity against Free Radicals 

Oxidative stress is defined as an imbalance between reactive oxygen species (ROS) and antioxidants. The excessive production of ROS may be associated with the pathogenesis of cancer, cardiovascular and neurological diseases [48]. Under physiological conditions, human RBCs are particularly vulnerable to oxidative stress when acting as oxygen transporters. In in vitro studies, the free radical generator 2,2′-azobis(2-methylpropionamidine) dihydrochloride (AAPH) is used to induce oxidative stress conditions [8,16]. In the current study, the cytoprotective activity of all complexes was assessed against AAPH-induced oxidative hemolysis.

**Figure 3:**
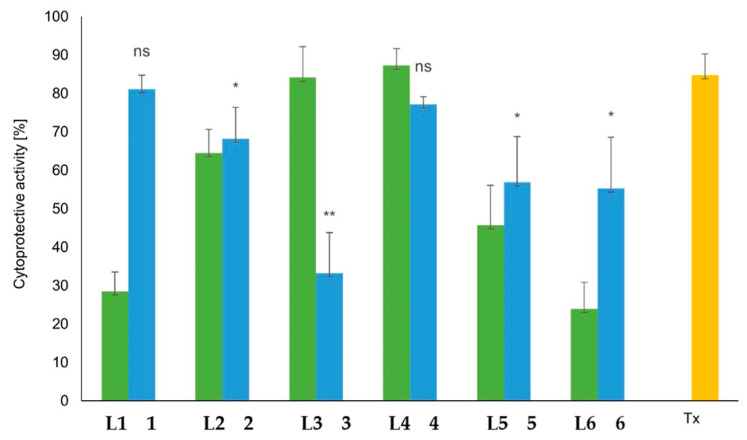
The cytoprotective activity of ligands **L1**–**L6** (green), their complexes with zinc chloride **1**–**6** (blue), and standard antioxidant Trolox (Tx) (orange) at the concentration of 0.1 mg/mL against AAPH-induced oxidative hemolysis. The results are presented as the mean value ± standard deviation. The activity of ligands is adopted from [16]. The activity of complexes is compared with Tx activity (* *p* < 0.05, ** *p* < 0.01). A non-statistically significant difference (*p* > 0.05) is indicated as ns.

As previously shown, the cytoprotective activity of indole–imidazole hybrids is strictly related to their structure [16]. The compounds with electron-donating groups in the imidazole ring showed the best inhibition of AAPH-induced hemolysis. In contrast, the compounds with electron-withdrawing substituents in imidazole showed an inhibition of AAPH-induced hemolysis in the range of 20% to 50% [16]. The cytoprotective activity of ligands and their complexes with zinc chloride at a concentration of 0.1 mg/mL is presented in Figure 3. 

In general, the cytoprotective activity of the complexes against AAPH-induced hemolysis was found to decrease in the order of **1** > **4** > **2** > **5** > **6** > **3**. The complexation of ligands **L1**, **L2**, **L5**, and **L6** increased their cytoprotective activity, with the most spectacular increase shown for **L1**, from 28.50% ± 5.00 to 81.15% ± 3.57. In addition, the activity of **1** was statistically the same as that of, Trolox, which is used as a standard antioxidant (*p* > 0.05). Complexes **3** and **4** were an exception because their cytoprotective activity was lower than that of the corresponding ligands. The possible reason for this difference is the presence of alkyl substituents (two methyl and two ethyl groups in complex **4**, and two isopropyl groups in complex **3**), totally hindering the approach to the metal center. This effect was the most spectacular for complex **3**, the cytoprotective activity of which decreased from 84.10% ± 8.00 (obtained for ligand) to 33.24% ± 10.54, respectively. In general, the size of the alkyl substituent played a significant role in reducing the cytoprotective activity of the complexes. 

A comparative analysis of the results obtained in the AAPH assay (Figure 3) and the molecular conformation of the crystals in complexes **1**–**5** (Figure 2) indicate that the high cytoprotective activity of compound **1** may be explained by its specific conformation, which facilitates interaction with the lipid bilayer of the RBC membrane. The incorporation of **1** into the RBC membrane may stabilize its molecular structure and ultimately increase the resistance of RBCs to ROS. 

### 2.4. Antibacterial Study

The bacteria used in this study are model species because they are widely distributed in nature. One of the most popular environmental species is *Bacillus subtilis*. These microorganisms develop best under aerobic conditions and can live in various temperatures, pH, and salinity environments [49]. *Micrococcus luteus* is a gram-positive bacterium found in soil, dust, water, and air, and is part of the normal microbiota of the mammalian skin. The bacterium also colonizes the human mouth, mucosae, oropharynx, and upper respiratory tract [50]. The best-known model bacterium is *Escherichia coli*. Some strains of *E. coli* can cause the development of many diseases in humans, causing mainly gastrointestinal infections [51]. *Pseudomonas fluorescens* is an aerobic gram-negative rod. It is commonly found in the air, water, soil, sewage, and plant tissues. In the case of human immunodeficiency, they can cause many diseases, causing respiratory and urinary tract infections, as well as the inflammation of many internal organs [52].

The tested complexes had a strong antagonistic effect on *Micrococcus luteus* bacteria. The strongest antagonistic response was observed for complex **1** when comparing the effects of the complexes with zinc chloride on the growth of the tested bacterial species (Table 3). The greatest sensitivity to this compound was shown by *M. luteus* (growth inhibition zone of 10.6 mm) and *E. coli* (5 mm). Complex **5** (with phenyl substituents) also showed strong antagonism, especially against *M. luteus* (growth inhibition zone of 8 mm) and *E. coli* (5 mm). These effects are related to the results obtained in tests of similar indole derivatives and their effects on bacterial cultures. Previous antibacterial studies have shown that analogous compounds that do not contain zinc chloride have the most significant inhibitory effect [16]. The weakest antibacterial activity was displayed by complexes **3** and **4**. In the case of complex **3**, no antagonistic reaction to *Pseudomonas fluorescens* culture was demonstrated. 

### 2.5. Fungicidal Activity

All the species of mold fungi used in the antifungal study constitute the environmental microbiome. *A. alternata* infects over 100 plant species, mainly their leaves and shoots [53]. *F. culmorum*, due to the production of many mycotoxins, can lead to severe food contamination [54]. The mold *B. cinerea* is a pathogen responsible for gray (or grey) mold diseases [55]. Both species of the *Trichoderma* genus are currently recognized as biological control agents and are used to combat many dangerous pathogens of crop plants in the natural environment [56]. 

The analysis of the fungistatic activity of the tested complexes showed that the development of fungi was most strongly inhibited by complex **6** (indole–benzimidazole ligands), especially in the genus *Trichoderma*, thus causing extensive zones of *T. harzianum* (11.5 mm) and *T. atroviride* (10 mm) population growth inhibition (Table 4). Complex **5** also had a strong inhibitory effect on mold growth, with the most significant inhibitory effect on *A. alternata* (10 mm). The lowest susceptibility of most molds was noted in the case of complexes **1** and **4**, except for *T. harzianum*, against which both complexes showed a strong antagonistic effect. An interesting effect of the tested complexes was observed in the response of *B. cinerea* cultures. In the case of this fungal species, not only did none of the tested compounds have an inhibitory effect, but the opposite phenomenon of stimulated development was observed where the tested compounds were introduced. The stimulation phenomenon was most pronounced in the case of complex **4**. The results presented are the diameter of the rim around the wells formed by the intensive development of the mold population. 

### 2.6. In Silico Study

The SwissADME website was used to calculate the physicochemical and pharmacokinetic properties of the complexes [57]. Solubility is one of the most important factors when designing new potential drugs. The better the solubility, the better the bioavailability, and the lower the dose required to reach therapeutic plasma concentrations after oral administration [58]. After this type of drug administration, the absorption of pharmaceuticals occurs mainly in the gastrointestinal (GI) tract, from where they enter the bloodstream and can then cross the blood–brain barrier (BBB) via passive diffusion [59].

All obtained complexes show worse physicochemical and pharmacokinetic properties than their ligands [16]. Table 5 shows that all complexes have a LogS range of −11–−7, which means that they are poorly soluble or completely insoluble in water. Complexes with imidazole and 2-methylimidazole ligands have the lowest partition coefficient (LogP) and are the least lipophilic; therefore, they have high GI absorption and can cross the blood–brain barrier. The remaining complexes, except **4**, have a low GI absorption and cannot cross the BBB. Complex **4** has a high GI absorption but cannot cross the BBB due to its lipophilic nature.

The high lipophilicity of complexes **5** and **6** (log P is 5.33 and 4.79, respectively) may explain their hemolytic activity.

## 3. Materials and Methods

### 3.1. Instrumentation and Chemicals

All melting points (mp) were obtained using a Büchi SMP-20 apparatus. The NMR (^1^H NMR, ^13^C NMR) was recorded in DMSO-*d*_6_ solution on a Varian 300/400 spectrometer (TMS as the internal standard). Chemical shifts are reported in δ (parts per million) values. The mass spectra (ESI–MS) were obtained using a ZQ Waters Mass Spectrometer. The IR spectra were recorded on a Nicolet iS 5 (KBr pellets). Elemental analysis was carried out by the means Elemental Analyzer Vario EL III, examining the percentage content of nitrogen, carbon and hydrogen. Analytical thin-layer chromatography (TLC) was carried out on silica gel plates 60 F254 (Sigma-Aldrich, Burlington, MA, USA) and visualized using UV. All chemicals or reagents used for syntheses were commercially available. In all reactions, anhydrous solvents were used. 

### 3.2. Synthesis of Gramine Derivatives

The synthesis of the indole–imidazole ligands (InIm) (**L1**), (InMeIm) (**L2**), (IniPrIm) (**L3**), (InEtMeIm) (**L4**), (InPhIm) (**L5**) and (InBzIm) (**L6**) was described in our previous paper [16].

A typical procedure for the synthesis of complexes **1**–**6**

Ligands (0.5 mmol) dissolved in 10 mL of absolute MeOH (for **L1**–**L5**) or CH_3_CN: MeOH 2:1 (for **L6**) were mixed with 0.025 mmol of ZnCl_2_ in 10 mL of MeOH and stirred for 2–5 h (**2**, **4**, **5**) or refluxed for 2 h (**1** and **3**). The mixtures were transferred to open vessels and left to crystallize. Products were obtained after 3–7 days, and only complexes **1** and **6** required recrystallization from MeOH.

[Zn(InIm)_2_Cl_2_] (**1**)

Where InIm is 3-((1H-imidazol-1-yl)methyl)-1H-indole

Colorless crystals (42 mg, 63%); m.p. 167–170 °C; ^1^H NMR (400 MHz, DMSO-*d*_6_): δ 11.20 (s, 1H), 8.19 (d, *J* = 1.3 Hz, 1H), 7.61–7.51 (m, 2H), 7.42–7.35 (m, 2H), 7.11 (ddd, *J* = 8.2, 7.0, 1.1 Hz, 1H), 7.06–6.94 (m, 2H), 5.43 (s, 2H); ^13^C NMR (101 MHz, DMSO-*d*_6_): δ 137.71, 136.22, 126.42, 125.99, 125.63, 121.58, 120.71, 119.16, 118.04, 111.77, 109.41, 42.23; IR (KBr): 3354, 3121, 3053, 2937, 1618, 1520, 1424, 1228, 1081, 747 cm^−1^; ESI–MS (ES^+^): 198 [M + H]^+^, 493 [2M-ZnCl]^+^, 553 [2M-ZnCl_2_-Na]^+^; Analysis calculated for C_24_H_22_Cl_2_N_6_Zn (MW = 530.76): C 54.31, H 4.18 and N 15.83; found: C 54.30, H 4.11 and N 16.13%.

[Zn(InMeIm)_2_Cl_2_] (**2**)

Where InMeIm is 3-((2-methyl-1H-imidazol-1-yl)methyl)-1H-indole

Colorless crystals (60 mg, 86%); m.p. 178–180 °C; ^1^H NMR (400 MHz, DMSO-*d*_6_) δ 11.21 (d, *J* = 2.6 Hz, 1H), 7.50 (dt, *J* = 8.0, 0.9 Hz, 1H), 7.46 (d, *J* = 2.6 Hz, 1H), 7.40 (dt, *J* = 8.1, 0.9 Hz, 1H), 7.28 (d, *J* = 1.6 Hz, 1H), 7.11 (ddd, *J* = 8.1, 7.0, 1.2 Hz, 1H), 7.00 (ddd, *J* = 8.0, 7.0, 1.0 Hz, 1H), 6.83 (d, *J* = 1.6 Hz, 1H), 5.30 (s, 2H), 2.45 (s, 3H); ^13^C NMR (101 MHz, DMSO-*d*_6_) δ 145.30, 136.29, 125.93, 125.35, 124.48, 121.59, 120.76, 119.15, 118.13, 111.82, 108.91, 41.69, 12.21; IR (KBr): 3359, 3133, 3061, 2981–2933, 1502, 1458, 1416, 1355, 1251, 751 cm^−1^; ESI–MS [ES]): 212 [M + H]^+^, 423 [2M]^+^, 521 [M-ZnCl]^+^; Analysis calculated for C_26_H_26_Cl_2_N_6_Zn (MW = 558.82): C 55.88, H 4.69 and N 15.04; found: C 55.56, H 4.68 and N 15.17%.

[Zn(IniPrIm)_2_Cl_2_] (**3**)

Where IniPrIm is 3-((2-isopropyl-1H-imidazol-1-yl)methyl)-1H-indole

Colorless crystals (55 mg, 71%); m.p. 190–193 °C; ^1^H NMR (400 MHz, DMSO-*d*_6_) δ 11.12 (s, 1H), 7.44 (d, *J* = 7.9 Hz, 1H), 7.42–7.34 (m, 2H), 7.15–7.06 (m, 2H), 6.99 (ddd, *J* = 8.0, 7.0, 1.1 Hz, 1H), 6.88 (s, 1H), 5.34 (s, 2H), 3.41 (m, 1H), 1.20 (d, *J* = 7.0 Hz, 6H); ^13^C NMR (101 MHz, DMSO-*d*_6_): δ 152.46, 136.30, 125.92, 125.35, 124.89, 121.49, 120.07, 119.02, 118.18, 111.74, 109.84, 41.47, 25.60, 21.26, 21.15; IR (KBr): 3680–3000, 2967, 1620, 1458, 1230, 1080, 735 cm^−1^; ESI–MS [ES]^+^: 240 [M − H]^+^, 577 [2M-ZnCl]^+^; Analysis calculated for C_30_H_34_C_l2_N_6_Zn (MW = 614.92): C 58.60, H 5.57 and N 13.67; found: C 58.26, H 5.40 and N 13.53%.

[Zn(InEtMeIm)_2_Cl_2_] (**4**)

Where InEtMeIm is 3-((2-ethyl-4-methyl-1H-imidazol-1-yl)methyl)-1H-indole

Colorless crystals (46 mg, 60%); m.p. 204–206 °C; ^1^H NMR (400 MHz, DMSO-*d*_6_) δ 11.18 (s, 1H), 7.48–7.45 (m, 1H), 7.40–7.37 (m, 1H), 7.10 (ddd, *J* = 8.1, 7.0, 1.2 Hz, 2H), 6.99 (tt, *J* = 8.2, 1.2 Hz, 2H), 6.84 (s, 1H), 5.22 (s, 2H), 2.98–2.87 (m, 2H), 1.99 (s, 3H), 1.09 (t, J = 7.5 Hz, 3H); ^13^C NMR (101 MHz, DMSO-*d*_6_): δ 150.23, 136.30, 133.68, 125.99, 125.07, 121.50, 119.06, 118.18, 117.91, 111.75, 109.67, 41.02, 19.02, 12.94, 9.38; IR (KBr): 3700–3150, 3120–2874, 1600, 1458, 1354, 1069, 748 cm^−1^; ESI–MS [ES]: 240 [M + H]^+^, 577 [2M-ZnCl]^+^, 635 [2M-ZnCl_2_-Na]^+^, 651 [2M-ZnCl_2_-K]^+^; Analysis calculated for C_30_H_34_Cl_2_N_6_Zn (MW = 614.92): C 58.60, H 5.57 and N 13.67; found: C 58.87, H 5.25 and N 13.72%.

[Zn(InPhIm)_2_Cl_2_] (**5**)

Where InPhIm is 3-((2-phenyl-2,5-dihydro-1H-imidazol-1-yl)methyl)-1H-indole

Light-brown crystals (133 mg, 50%); m.p. 189–191 °C; ^1^H NMR (400 MHz, DMSO-*d*_6_) δ 11.13 (s, 1H), 7.62 (dd, *J* = 6.9, 2.1 Hz, 2H), 7.55–7.40 (m, 3H), 7.36 (dt, *J* = 8.3, 1.0 Hz, 2H), 7.21–7.03 (m, 4H), 6.93 (ddd, *J* = 8.0, 7.0, 1.0 Hz, 1H), 5.35 (s, 2H); ^13^C NMR (101 MHz, DMSO-*d*_6_): δ 146.99, 136.14, 129.63, 129.55 (2×), 128.51 (3×), 126.68, 125.71, 124.93, 121.75, 121.57, 119.11, 117.79, 111.77, 109.43, 42.58; IR (KBr): 3750–3000, 1620, 1428, 1358, 1233, 747 cm^−1^; ESI–MS [ES]^+^: 274 [M]^+^, 547 [2M]^+^, 649 [2M-ZnCl]^+^; Analysis calculated for C_36_H_30_Cl_2_N_6_Zn (MW = 682.96): C 63.31, H 4.43 and N 12.31; found: C 63.68, H 4.24 and N 12.46%.

[Zn_2_(InBzIm)_2_Cl_2_] (**6**)

Where InBzIm is 1-((1H-indol-3-yl)methyl)-1H-benzo[d]imidazole

Colorless crystals (56 mg, 71%); m.p. 207–209 °C ^1^H NMR (400 MHz, DMSO-*d*_6_) δ 11.18 (s, 1H), 8.78 (s, 1H), 7.83–7.78 (m, 1H), 7.74 (d, *J* = 8.1 Hz, 1H), 7.67 (d, *J* = 2.6 Hz, 1H), 7.57 (ddt, *J* = 7.9, 1.3, 0.7 Hz, 1H), 7.36 (dt, *J* = 8.1, 0.9 Hz, 1H), 7.29 (ddd, *J* = 8.2, 7.2, 1.2 Hz, 1H), 7.21 (ddd, *J* = 8.2, 7.2, 1.2 Hz, 1H), 7.08 (ddd, *J* = 8.2, 7.0, 1.2 Hz, 1H), 6.96 (ddd, *J* = 8.0, 7.0, 1.0 Hz, 1H), 5.73 (s, 2H); ^13^C NMR (101 MHz, DMSO-*d*_6_): δ 144.49, 140.81, 136.30, 133.06, 125.97, 125.78, 123.20, 122.72, 121.50, 119.09, 118.40, 118.12, 111.81, 111.78, 108.80, 40.56; IR (KBr): 3641, 3640–2920, 1638, 1491, 1343, 1265, 753 cm^−1^; ESI–MS [ES]^+^: 248 [M + H]^+^, 593 [2M-ZnCl]^+^, 842 [3M-ZnCl]^+^; Analysis calculated for C_32_H_26_Cl_2_N_6_Zn (MW = 630.88): C 60.92, H 4.15 and N 13.32; found: C 61.02, H 3.92 and N 13.62%.

### 3.3. X-ray Analysis

Single-crystal X-ray diffraction measurements were carried out with the monochromated MoK_α_ radiation on an Xcalibur diffractometer. The data were collected and processed using the CrysAlis^Pro^ software [60]. The crystal structures were solved using direct methods with SHELXT [61] and refined using full-matrix least-squares calculations on F^2^ with SHELXL [62]. All non-H atoms were refined using anisotropic displacement parameters. Hydrogen atoms were placed at calculated positions based on the environment and the perceived hybridization of the C atoms to which they are bonded (methyl C–H = 0.96 Å, methylene C–H = 0.97 Å, aromatic C-H = 0.93 Å and N-H = 0.86 Å); they were then refined as ‘riding’ on their carriers. During the refinement, isotropic displacement parameters for H-atoms were assigned 20% higher than the isotropic equivalent for the atom to which the H-atom was bonded. The crystals of **3** were twinned along the x-axis. Moreover, in the crystals of **3** and **4**, and in **5** of the terminal phenyl groups, there were signs of disorder in the indole moiety. We have made no attempts to model this disorder. Some atoms in these three structures were restrained so that their U_ij_ components were approximate to isotropic behavior using an ISOR command incorporated into the SHELX program. The final model is not fully satisfactory, as some of the atomic displacement parameters for the atoms involved in the disorder are relatively high and the C–C bonds to these atoms are determined with low precision. MERCURY [47] computer graphics programs were used to prepare drawings. The crystal data, together with the experimental and refinement details, are collected in Table 6. CCDC 2259536-2259540 contains the supplementary crystallographic data for this paper (for structures **1–5**, respectively). These data can be obtained free of charge via http://www.ccdc.cam.ac.uk/conts/retrieving.html (or from the CCDC, 12 Union Road, Cambridge CB2 1EZ, UK; Fax: +44-1223-336033; E-mail: deposit@ccdc.cam.ac.uk).

### 3.4. Biological Activity 

#### 3.4.1. Human Erythrocyte

All experimental procedures were conducted in accordance with the relevant guidelines and regulations, and were approved by the Bioethics Committee for Scientific Research at the Medical University in Poznań (approval number ZP/2867/D/21). All human red blood cell (RBC) concentrates used in this study were purchased from the Blood Bank in Poznań, and there was no direct contact with blood donors.

The RBC suspensions were washed three times (10 min at 3000 rpm, +4 °C) using 7.4 pH phosphate-buffered saline (PBS) supplemented with 10 mM glucose (PBS: 137 mM NaCl, 2.7 mM KCl, 10 mM Na_2_HPO_4_, 1.76 mM KH_2_PO_4_). Following the washing process, the RBCs were resuspended in the PBS buffer at a concentration of 1.65 × 10^9^ cells/mL, stored at +4 °C, and utilized within 5 h.

#### 3.4.2. Hemolytic Activity

To determine the cytotoxic effects of the tested compounds, a standard hemolytic assay was performed according to the protocol of Mrówczyńska and Hägerstrand [63]. In summary, RBCs were resuspended in a phosphate-buffered saline (PBS) at pH 7.4 and supplemented with 10 mM of glucose. The RBC concentration was adjusted to 1.65 × 10^8^ cells/mL, with a hematocrit of 1.5%. RBCs were then incubated with the tested compounds at a concentration of 0.1 mg/mL for 60 min at 37 °C under shaking. A negative control was prepared by incubating RBCs in PBS buffer without the test compounds, and a positive control was prepared by incubating RBCs in ice-cold water. Each sample was made in triplicate, and experiments were performed three times using RBCs obtained from different blood donors. Following the incubation, RBC suspensions were centrifuged at 3000 rpm for 10 min (4 °C), and the degree of hemolysis was determined by measuring the absorbance (Ab) of the supernatant at 540 nm. The results were presented as the percentage (%) of hemolysis, which was calculated using the following formula:

Hemolysis% = (Ab_sample_/Ab_positive control_) × 100

Data were presented as the mean value ± standard deviation (SD) (n = 9).

#### 3.4.3. Protective Activity against Oxidative Stress-Induced Hemolysis

To assess the effects of compounds on free radical-induced hemolysis, RBCs (1.65 × 10^8^ cells/mL, with a hematocrit of 1.5%) were preincubated with the tested compounds (0.1 mg/mL) in a phosphate-buffered saline (PBS) solution supplemented with 10 mM of glucose for 20 min at 37 °C with shaking. After preincubation, 2,2’-azobis(2-methylpropionamidine) dihydrochloride (AAPH) was added to a final concentration of 60 mM, and samples were incubated for an additional 4 h at 37 °C with shaking. A negative and a positive control were included, consisting of RBCs incubated in PBS and in the presence of AAPH, respectively. Following incubation, RBC suspensions were centrifuged at 4000 rpm for 5 min at 4 °C, and the absorbance of the supernatants was measured at 540 nm using a spectrophotometer. The absorbance values obtained were used to calculate the cytoprotective activity of the tested compounds using the following formula:

Cytoprotective activity (%) = 100 − [(Ab_sample_/Ab_AAPH_) × 100]

where Ab_sample_ is the absorbance of the supernatant obtained from samples incubated with compounds tested in the presence of AAPH, and Ab_AAPH_ is the absorbance of the supernatant obtained using AAPH controls (without tested compounds). Each sample was tested in triplicate and in three independent experiments using RBCs obtained from different blood donors. 

Data are presented as the mean value ± standard deviation (SD) (n = 9).

#### 3.4.4. Statistical Analysis 

A paired *t*-Student test was conducted to compare the activity of the tested derivatives with that of the standard antioxidant Trolox. Statistical significance was defined as *p* < 0.05, and non-significant differences were indicated as “ns”.

### 3.5. Antibacterial and Antifungal Activity Measurements 

The effects of the compounds on microorganisms were tested on the following bacterial strains: *Micrococcus luteus*, *Bacillus subtilis*, *Escherichia coli*, and *Pseudomonas fluorescens*. The antifungal activity of the compounds was determined against *Alternaria alternata*, *Fusarium culmorum*, *Trichoderma harzianum*, *Trichoderma harzianum*, and *Botrytis cinerea*. All cultures of microorganisms came from the collection of Pure Cultures of the Facility of Microbiology of the Department of Soil Science and Microbiology of the Poznan University of Life Sciences. Bacteria were cultured on a broth medium, while mold fungi were grown on potato dextrose agar (PDA). The well diffusion method was used to evaluate the antimicrobial properties of the compounds. Then, 6 mL of appropriate culture media was poured onto Petri dishes in order to stabilize the glass rings, which were placed on the solidified layer of the medium. Two glass rings with a diameter of 0.5 cm were placed on each plate. Then, 20 mL of each liquid medium containing suspensions of the tested microorganisms was introduced. The final suspension of bacteria had a density of 107 cells/cm^3^, obtained from 48-h cultures on broth slants, and the fungal suspension had a density of 108 spores/cm^3^, obtained from 5-day cultures on the PDA slants. The rings, after their removal, obtained two wells on each plate. One well was filled with 0.1 mL of the tested compound dissolved in dimethyl sulfoxide, while the other was filled with only 0.1 mL of dimethyl sulfoxide, which was a control.

Each compound was tested in four replicates. The plates were incubated for 48 h in a thermostat at 27 °C for *M. luteus*, *B. subtilis* and *P. fluorescens* cultures, and the *E. coli* culture was incubated at 37 °C. All fungal cultures were incubated for 72 h in a thermostat at 24 °C. After the strains were cultured on Petri dishes, the zones of microorganism growth inhibition were observed around the wells containing the tested compounds. The width of these zones was measured accurately using calipers.

### 3.6. In Silico Study

The physicochemical calculations were conducted using the SwissADME website: www.swissadme.ch (accessed on 23 March 2023).

## 4. Conclusions

This work aimed to combine Zn(II) with hybrid heterocyclic ligands that are known for their biological activity in order to potentially enhance their function. Therefore, we undertook synthetic, spectroscopic, structural and biological activity studies in order to verify whether this is, indeed, the case. The obtained results show that the cytoprotective activity of the indole–imidazole ligand significantly increases upon its complexation with ZnCl_2_ up to a value comparable with the standard antioxidant Trolox. Some increase is also observed in the case of the indole–benzimidazole hybrid. With regard to the biological function of the ligands containing the substituted imidazole ring, it does not change significantly upon complexation with ZnCl_2_, except for the derivative that has an isopropyl group, for which it considerably diminishes. Remarkably, this molecule is the most structurally affected by complexation. Moreover, in contrast to the other complexes that associate into polar assemblies, it forms centrosymmetric supramolecular aggregates. Molecular association is mostly governed by the N-H(indole)···Cl(chloride) intermolecular hydrogen bonds, and the supramolecular assemblies that are thus formed differ, depending on a distinctive molecular shape, which can be compact (as in **1** and **2**) or extended (as in **4** and **5**). 

## Figures and Tables

**Table 1:** Torsion angles (◦) describing the rotation around the methylene C–C (φ_1_) and C–N (φ_2_) bonds in Zn(II)-complexed hybrid indole–imidazole ligands, as present in crystals. Where available, these values have been compared to those of the uncomplexed ligands [16]. The angle φ_3_ describes the twist of one of the imidazole rings around the Zn–N coordination bond with respect to the other ring.

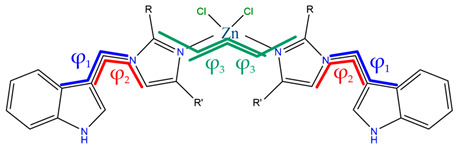				
		**φ_1_**	**φ_2_**	**φ_3_**
**1** *	R=H, R’=H	86.7 (4)	−79.1 (4)	−46.0 (4)
**2** *	R=Me, R’=H	97.5 (3)	−87.2 (3)	−47.0 (3)
**3**	R=iPr R’=H	88.9 (5)	−14.5 (7)	59.9 (6)
		71.9 (5)	18.1 (6)	54.7 (6)
**L3**	R=iPr, R’=H	−65.39 (17)	−34.9 (2)	
**4** *	R=Et, R’=Me	81.6 (8)	59.2 (7)	−66.8 (7)
**5**	R=Ph, R’=H	68.7 (5)	41.8 (5)	−49.0 (5)
		68.1 (6)	46.1 (6)	−41.6 (6)
**L5**	R=Ph, R’=H	−62.47 (19)	−31.3 (2)	

* *C*_2_ molecular symmetry.

**Table 2:** Hydrogen bond parameters.

*D*—H···*A*	*D*—H (Å)	H···*A* (Å)	*D*···*A* (Å)	*D*—H···*A* (°)
**1**				
N1—H1···Cl1 ^i^	0.86	2.45	3.260 (3)	157
**2**				
N1—H1···Cl1 ^i^	0.86	2.58	3.345 (2)	148
**3**				
N1—H1···Cl1 ^ii^	0.86	2.47	3.324 (3)	170
N21—H21···Cl2 ^iii^	0.86	2.68	3.398 (4)	142
**4**				
N1—H1···Cl1 ^iv^	0.86	2.57	3.373 (7)	156
**5**				
N1—H1···Cl2 ^v^	0.86	2.49	3.337 (3)	167
N21—H21···Cl1 ^vi^	0.86	2.40	3.236 (3)	165

Symmetry code(s): (^i^) −*x* + 1, −*y* + 1, *z* + 1; (^ii^) −*x* + 1, −*y* + 1, −*z* + 2; (^iii^) −*x* + 2, −*y*, −*z* + 1; (^iv^) −*x* + 1/2, *y* − 1/2, −*z* + 3/2; (^v^) −*x* + 1, *y* + 1/2, −*z* + 1/2; (^vi^) −*x* + 2, *y* + 1/2, −*z* + 3/2.

**Table 3:** Antibacterial activities of complexes **1**–**6** (5 mg/mL).

Complex	Zone of Growth Inhibition [mm]			
	*Micrococus* *luteus*	*Bacillus* *subtilis*	*Escherichia coli*	*Pseudomonas* *fluorescens*
**1**	10.6	4	5	3
**2**	6	4	4.3	3.4
**3**	3.7	3	3.5	0
**4**	2.4	2.3	3.7	3.3
**5**	8	4	5	4
**6**	7.6	4.8	4	4

**Table 4:** Antifungal activity of complexes **1**–**6** (5 mg/mL).

Complex	Zone of Growth Inhibition [mm]				Zones of Growth Stimulation [mm]
	*Alternaria alternata*	*Fusarium* *culmorum*	*Trichoderma harzianum*	*Trichoderma atroviride*	*Botrysis* *cinera*
**1**	4.5	0	10	0	13.3
**2**	7.5	2	3	4	16.8
**3**	4.5	6	10	2	17
**4**	2.5	0	11.5	2	21.2
**5**	10	6.4	9.8	2	15.3
**6**	7.5	9.2	11.5	10	11.7

**Table 5:** LogP, GI absorption, BBB permeability and water solubility of complexes **1**–**6**, calculated in SwissADME. LogS in the table is the average value of logS calculated using three different methods. * Solubility class—logS scale: Insoluble < −10, Poorly < −6, Moderately < −4, Soluble < −2, Very < 0.

Compound	LogP	GI Absorption	BBB Permeant	LogS	Solubility Class *
**1**	2.91	High	Yes	−7.16	Poorly soluble
**2**	3.48	High	Yes	−7.89	Poorly soluble
**3**	4.52	Low	No	−9.03	Poorly soluble
**4**	4.57	High	No	−9.38	Poorly soluble
**5**	5.33	Low	No	−10.9	Insoluble
**6**	4.79	Low	No	−10.03	Insoluble

**Table 6:** Crystal data and structure refinement parameters for complexes **1**–**5**. The measurements were carried out at 295 K with Mo *K*α radiation using a Xcalibur diffractometer equipped with an Eos detector. H-atom parameters were constrained.

	1	2	3	4	5
Chemical formula	C_24_H_22_Cl_2_N_6_Zn	C_26_H_26_Cl_2_N_6_Zn	C_30_H_34_Cl_2_N_6_Zn	C_30_H_34_Cl_2_N_6_Zn	C_36_H_30_Cl_2_N_6_Zn
*M*_r_	530.74	558.80	614.90	614.90	682.93
Crystal system, space group	Orthorhombic, *Fdd*2	Orthorhombic, *Fdd*2	Triclinic, *P*$\overset{-}{1}$	Monoclinic, *C*2/*c*	Monoclinic, *P*2_1_/*c*
*a* (Å) *b* (Å) *c* (Å)	25.6543 (9) 18.1710 (6) 10.4135 (3)	30.2233 (5) 16.0385 (2) 10.5687 (1)	8.6293 (5) 12.5629 (7) 15.3951 (13)	21.6193 (4) 9.0501 (2) 15.1471 (3)	16.9185 (9) 10.2496 (3) 19.3748 (6)
α (°) β (°) γ (°)	90 90 90	90 90 90	78.332 (6) 76.354 (6) 71.776 (5)	90 93.383 (2) 90	90 105.642 (4) 90
*V* (Å^3^)	4854.4 (3)	5123.03 (12)	1525.51 (19)	2958.47 (10)	3235.3 (2)
*Z*	8	8	2	4	4
*D_x_* (Mg m^−3^)	1.452	1.449	1.339	1.381	1.402
μ (mm^−1^)	1.26	1.19	1.01	1.04	0.96
Crystal size (mm)	0.25 × 0.18 × 0.15	0.60 × 0.20 × 0.12	0.40 × 0.25 × 0.25	0.35 × 0.35 × 0.05	0.35 × 0.23 × 0.03
Data collection					
*T*_min_, *T*_max_	0.955, 1.000	0.901, 1.000	0.973, 1.000	0.830, 1.000	0.962, 1.000
No. of measured, independent and observed [*I* > 2θ(*I*)] reflections	18,213, 2834, 2425	19,144, 2957, 2773	14,779, 14,779, 7369	39,542, 3485, 2193	38,365, 7181, 3683
*R*_int_	0.024	0.017	–	0.035	0.063
(sin θ/λ)_max_ (Å^−1^)	0.670	0.672	0.674	0.667	0.667
Refinement					
*R*[*F*^2^ > 2σ(*F*^2^)], *wR*(*F*^2^), *S*	0.028, 0.062, 1.03	0.021, 0.057, 1.09	0.046, 0.123, 0.87	0.077, 0.239, 1.03	0.063, 0.135, 1.02
No. of reflections	2834	2957	14,779	3485	7181
No. of parameters	150	160	357	179	406
No. of restraints	1	1	36	30	12
Δρ_max_, Δρ_min_ (e Å^−3^)	0.21, −0.17	0.24, −0.15	0.59, −0.32	1.04, −0.39	0.63, −0.32
Absolute structure parameter	0.003 (4)	−0.013 (3)	–	–	–

## Data Availability

Not applicable.

## Supplementary Materials

The following supporting information can be downloaded at: https://www.mdpi.com/article/10.3390/molecules28104132/s1, Figure S1a: ^13^C NMR spectrum (101 MHz, DMSO-*d*_6_): complex **1**; Figure S1b: ^1^H NMR spectrum (400 MHz, DMSO-*d*_6_): complex **1**; Figure S2: IR spectrum: complex **1**; Figure S3: ESI–MS spectrum: complex **1**; Figure S4a: ^13^C NMR spectrum (101 MHz, DMSO-*d*_6_): complex **2**; Figure S4b: ^1^H NMR spectrum (400 MHz, DMSO-*d*_6_): complex **2**; Figure S5: IR spectrum: complex **2**; Figure S6: ESI–MS spectrum: complex **2**; Figure S7a: ^13^H NMR spectrum (101 MHz, DMSO-*d*_6_): complex **3**; Figure S7b: ^1^H NMR spectrum (400 MHz, DMSO-*d*_6_): complex **3**; Figure S8: IR spectrum: complex **3**; Figure S9: ESI–MS spectrum: complex **3**; Figure S10a: ^13^H NMR spectrum (101 MHz, DMSO-*d*_6_): complex **4**; Figure S10b: ^1^H NMR spectrum (400 MHz, DMSO-*d*_6_): complex **4**; Figure S11: IR spectrum: complex **4**; Figure S12: ESI–MS spectrum: complex **4**; Figure S13a: ^13^H NMR spectrum (101 MHz, DMSO-*d*_6_): complex **5**; Figure S13b: ^1^H NMR spectrum (400 MHz, DMSO-*d*_6_): complex **5**; Figure S14: IR spectrum: complex **5**; Figure S15: ESI–MS spectrum: complex **5**; Figure S16a: ^13^C NMR spectrum (101 MHz, DMSO-*d*_6_): complex **6**; Figure S16b: ^1^H NMR spectrum (400 MHz, DMSO-*d*_6_):complex **6**; Figure S17: IR spectrum: complex **6**; Figure S18: ESI–MS spectrum: complex **6**; Table S1: Selected bond lengths and angles for complexes **1**–**5**.

## Sample Availability

Samples of the compounds are not available from the authors.
