# Supplementary material for: Synthesis, Structure and Biological Activity of Indole–Imidazole Complexes with ZnCl2: Can Coordination Enhance the Functionality of Bioactive Ligands?
Source: Molecules. 2023 May 16;28(10):4132. doi: 10.3390/molecules28104132 (PMC10220783; doi:10.3390/molecules28104132)
Supplement: Supplementary file 1 [file molecules-28-04132-s001.zip › molecules-2398318-supplementary.pdf]

Supplementary data

Synthesis, structure and biological activity of indole-imidazole complexes with $ZnCl_2$: can coordination enhance functionality of bioactive ligands?

Karolina Babijczuk[1], Beata Warżajtis[2], Justyna Starzyk[3], Lucyna Mrówczyńska[4], Beata Jasiewicz[1*] and Urszula Rychlewska[2*]

[1]Department of Bioactive Products, Faculty of Chemistry, Adam Mickiewicz University, Uniwersytetu Poznańskiego 8, 61-614 Poznań, Poland,

[2]Department of Crystallography, Faculty of Chemistry, Adam Mickiewicz University, Uniwersytetu Poznańskiego 8, 61-614 Poznań, Poland,

[3]Department of Soil Science and Microbiology, Faculty of Agronomy, Horticulture, and Bioengineering, University of Life Science, Szydłowska 50, 60-656 Poznań, Poland,

[4]Department of Cell Biology, Faculty of Biology, Adam Mickiewicz University, Uniwersytetu Poznańskiego 6, 61-614 Poznań, Poland,

TABLE OF CONTENTS

^{1}H and ^{13}C NMR spectra of complex **1**……S2

IR and ESI-MS spectra of complex **1**……S3

^{1}H and ^{13}C NMR spectra of complex **2**……S4

IR and ESI-MS spectra of complex **2**……S5

^{1}H and ^{13}C NMR spectra of complex **3**……S6

IR and ESI-MS spectra of complex **3**……S7

^{1}H and ^{13}C NMR spectra of complex **4**……S8

IR and ESI-MS spectra of complex **4**……S9

^{1}H and ^{13}C NMR spectra of complex **5**……S10

IR and ESI-MS spectra of complex **5**……S11

^{1}H and ^{13}C NMR spectra of complex **6**……S12

IR and ESI-MS spectra of complex **6**……S13

Table S1. Selected bond lengths and angles for complexes **1-5**……S14

(a)

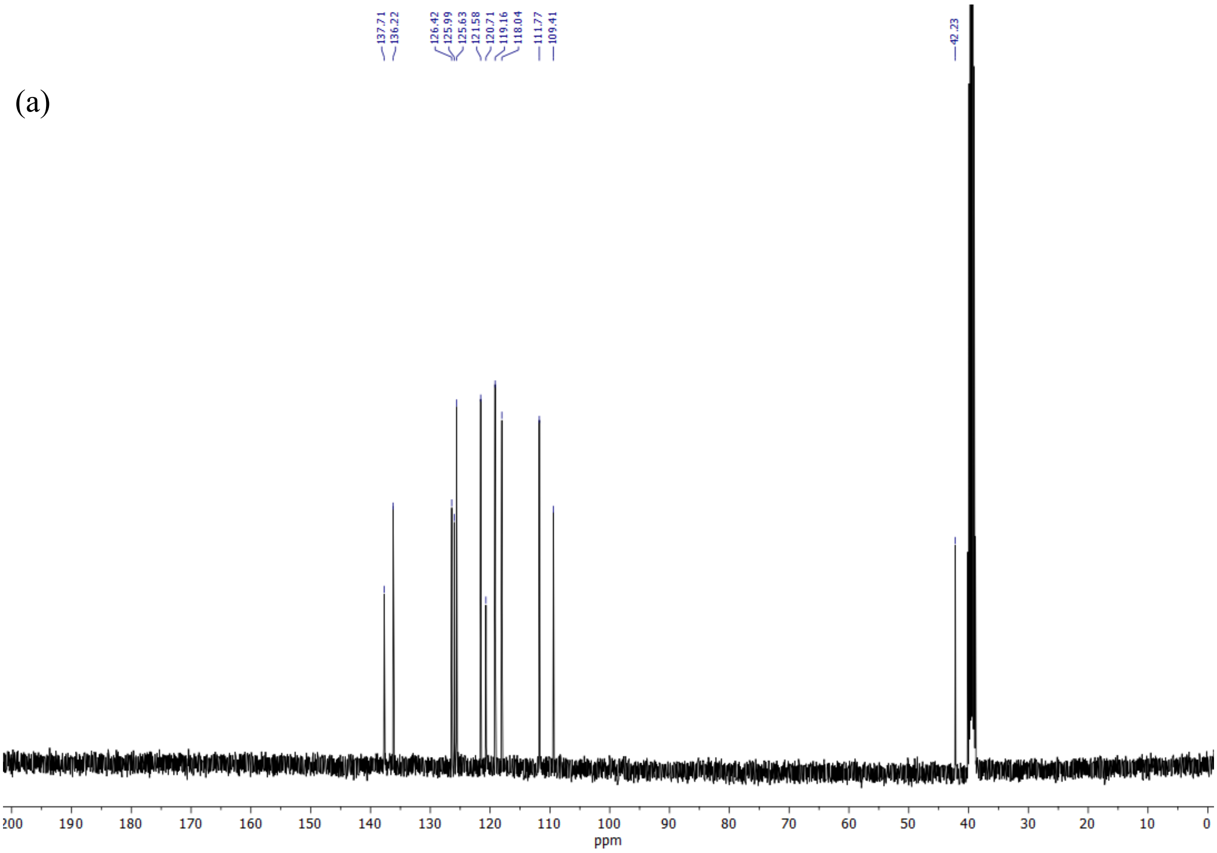

(b)

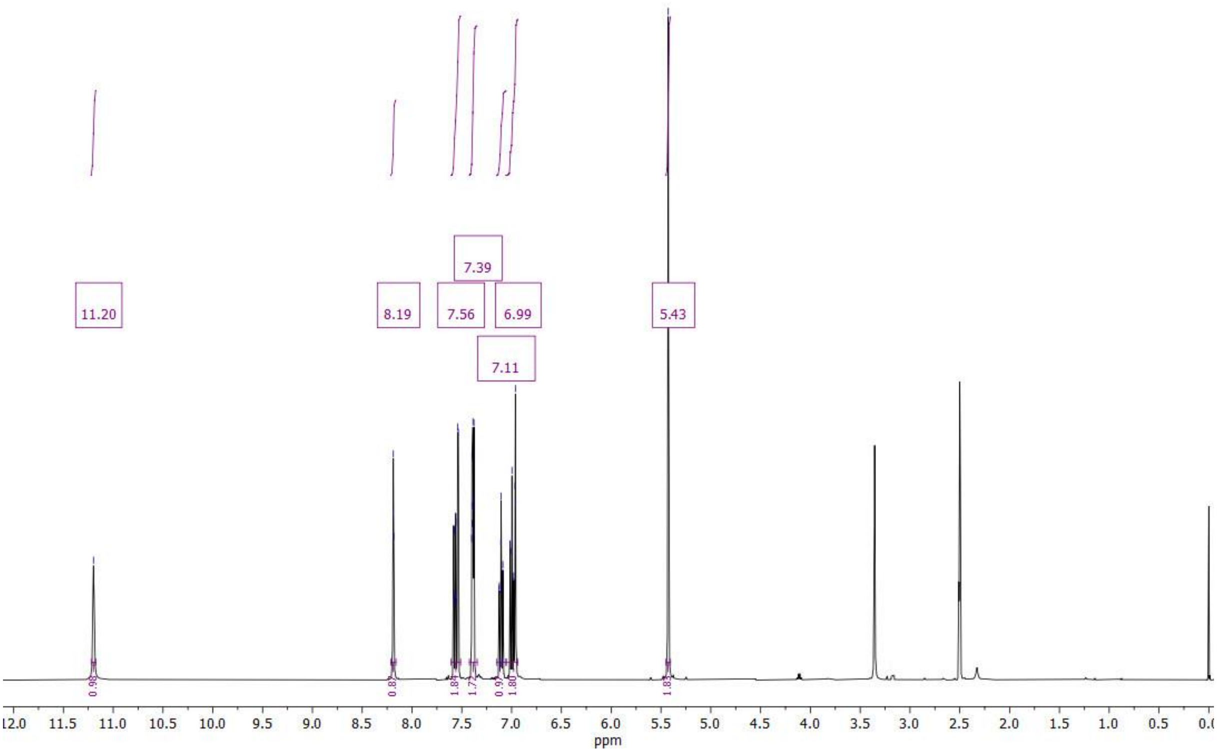

Figure S1 (a) 13C NMR spectrum of complex **1** **(b)** ^{1}H NMR spectrum of complex **1**

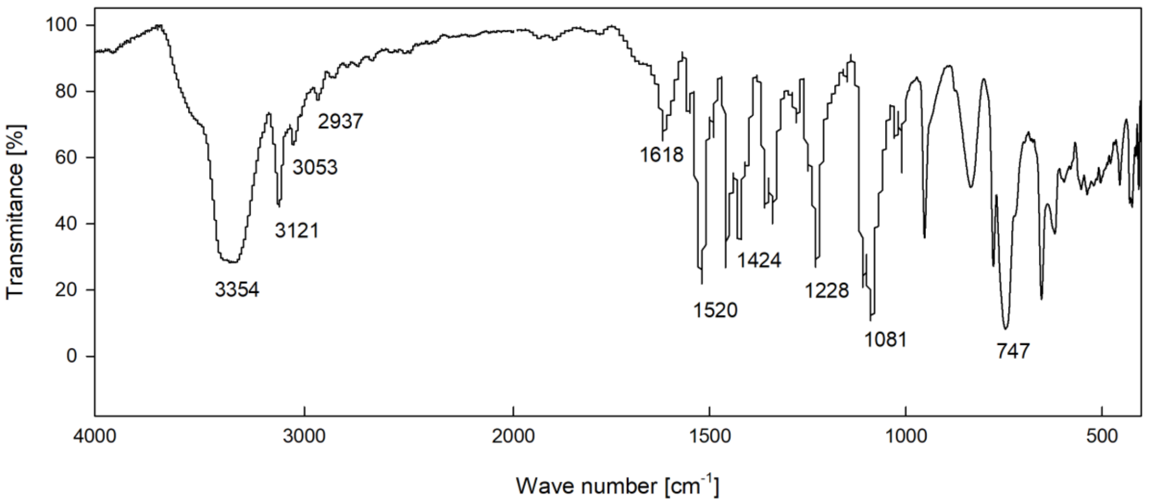

Figure S2. IR spectrum of complex **1**

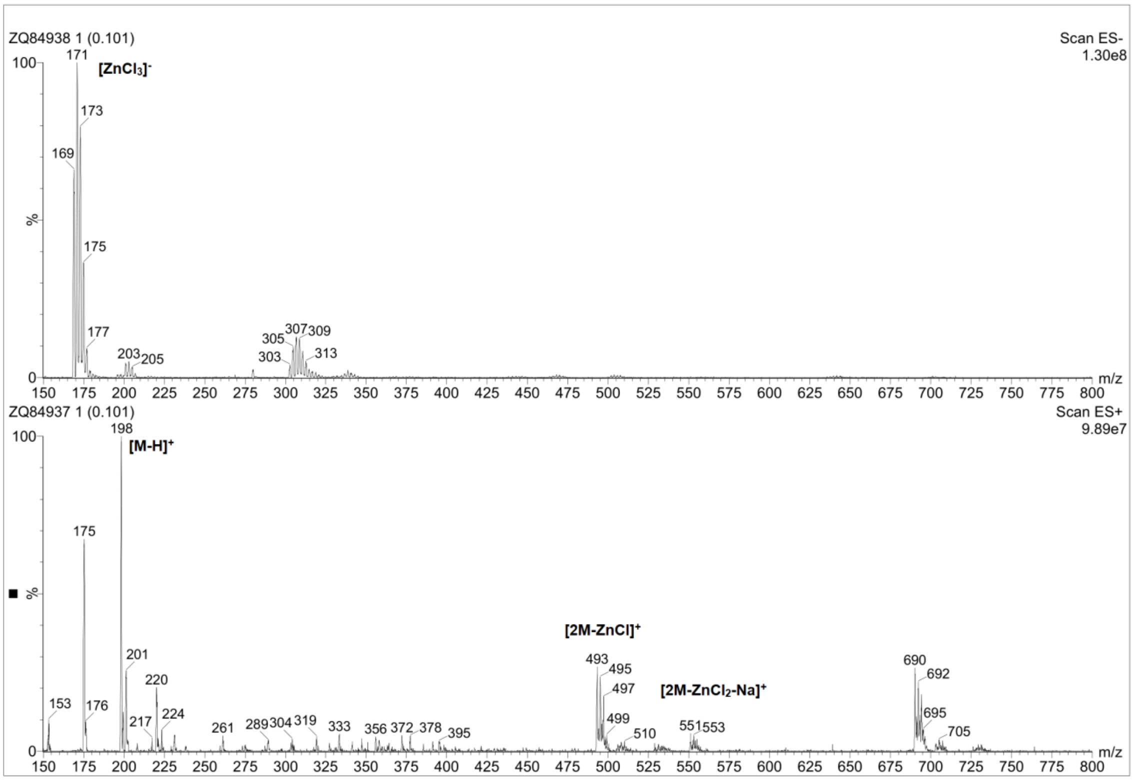

Figure S3. ESI-MS spectrum of complex **1**

(a)

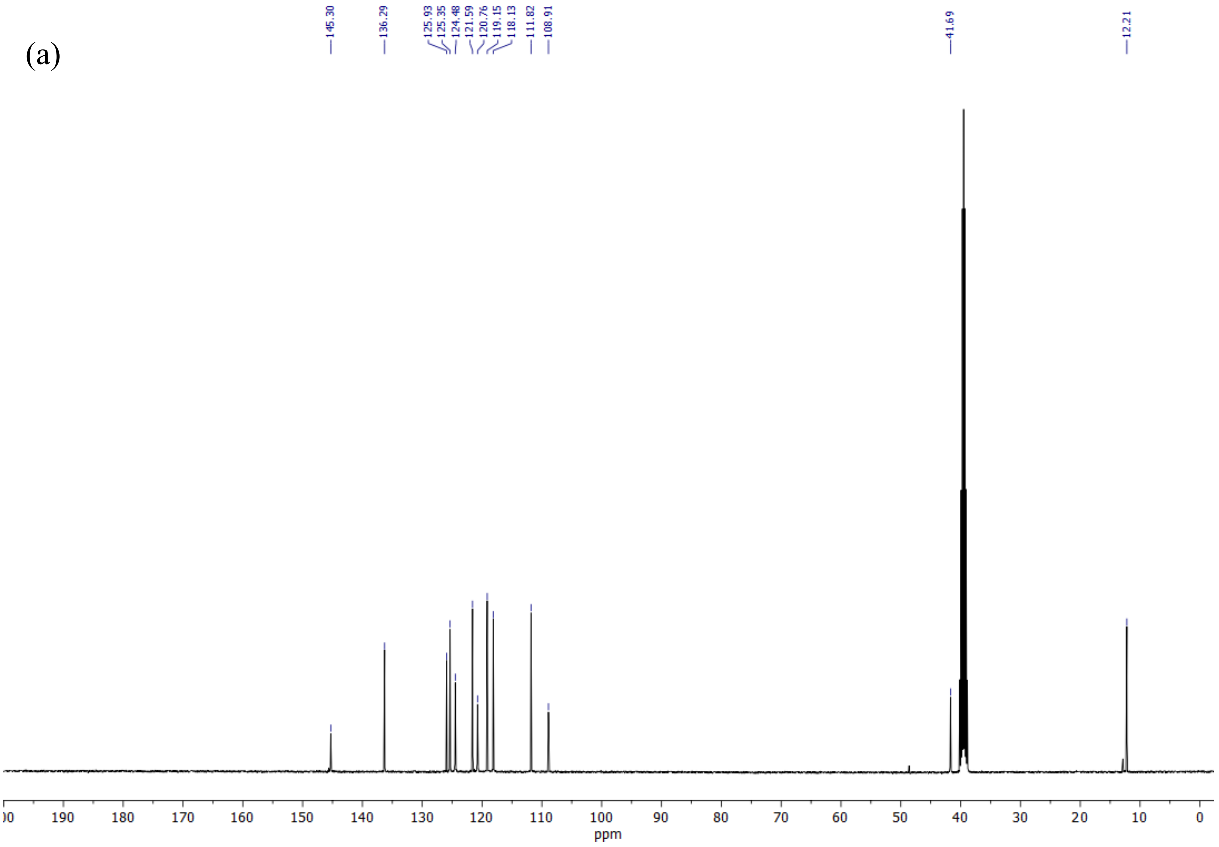

(b)

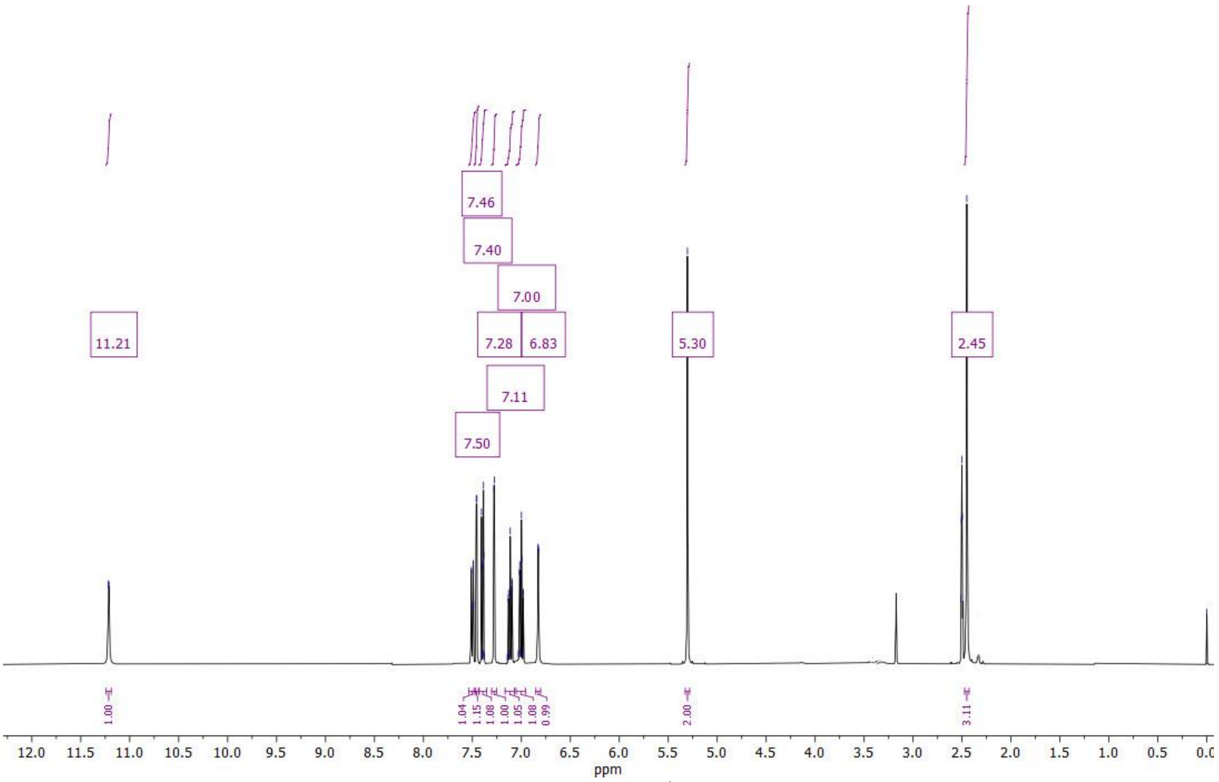

Figure S4 (a) ^{13}C NMR spectrum of complex **2** **(b)** ^{1}H NMR spectrum of complex **2**

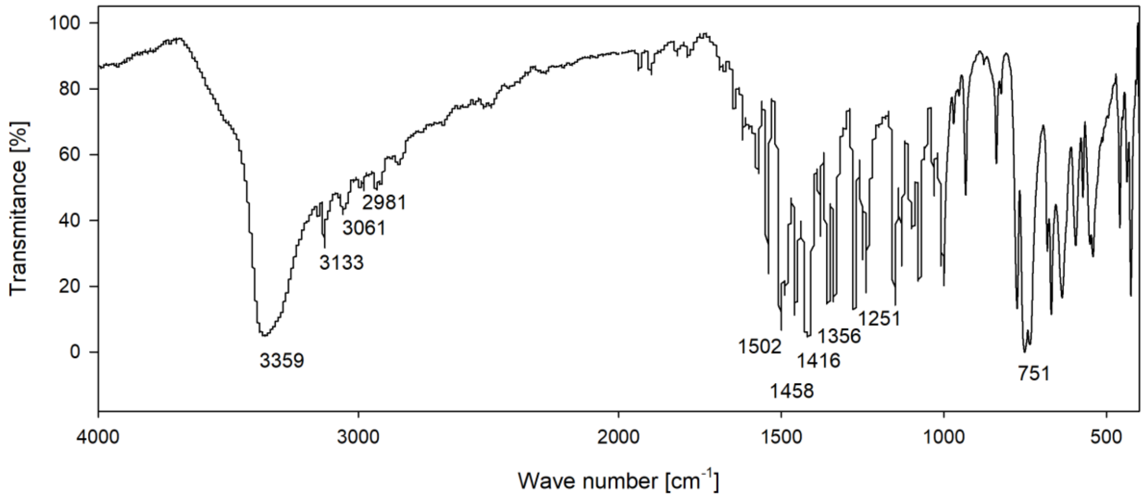

Figure S5. IR spectrum of complex **2**

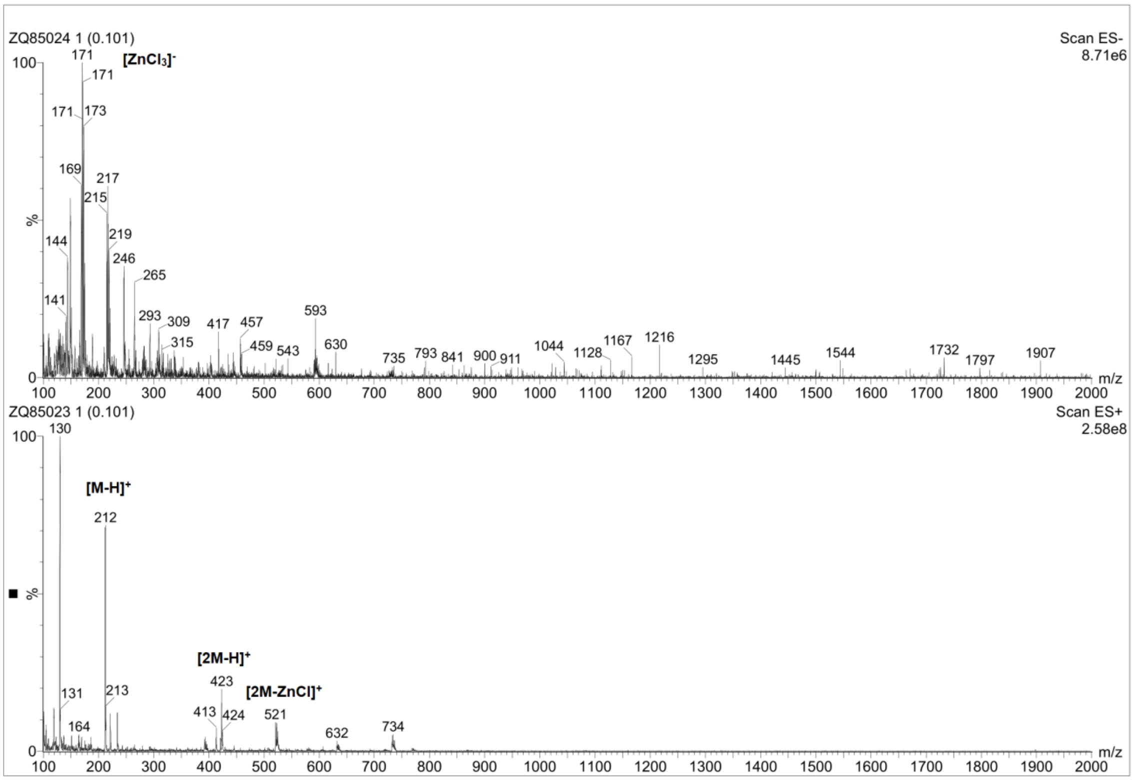

Figure S6. ESI-MS spectrum of complex **2**

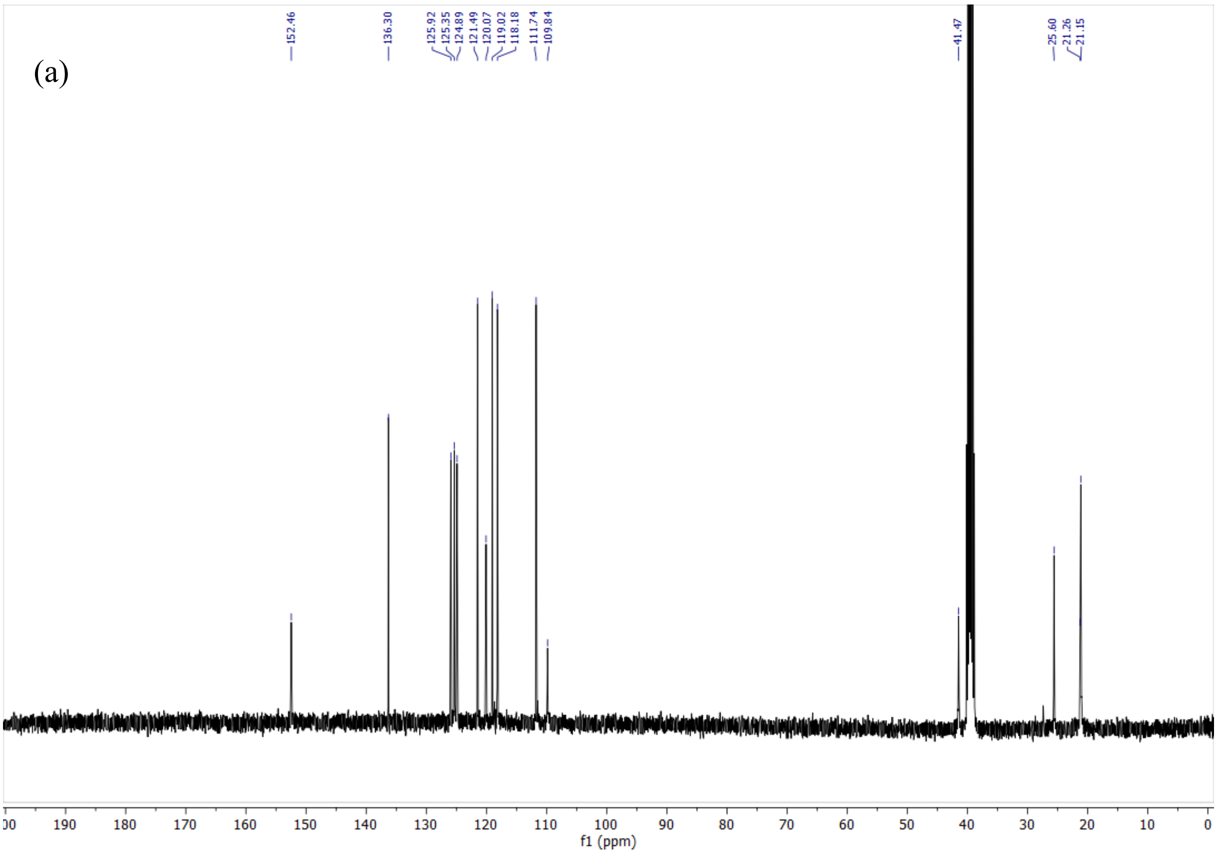

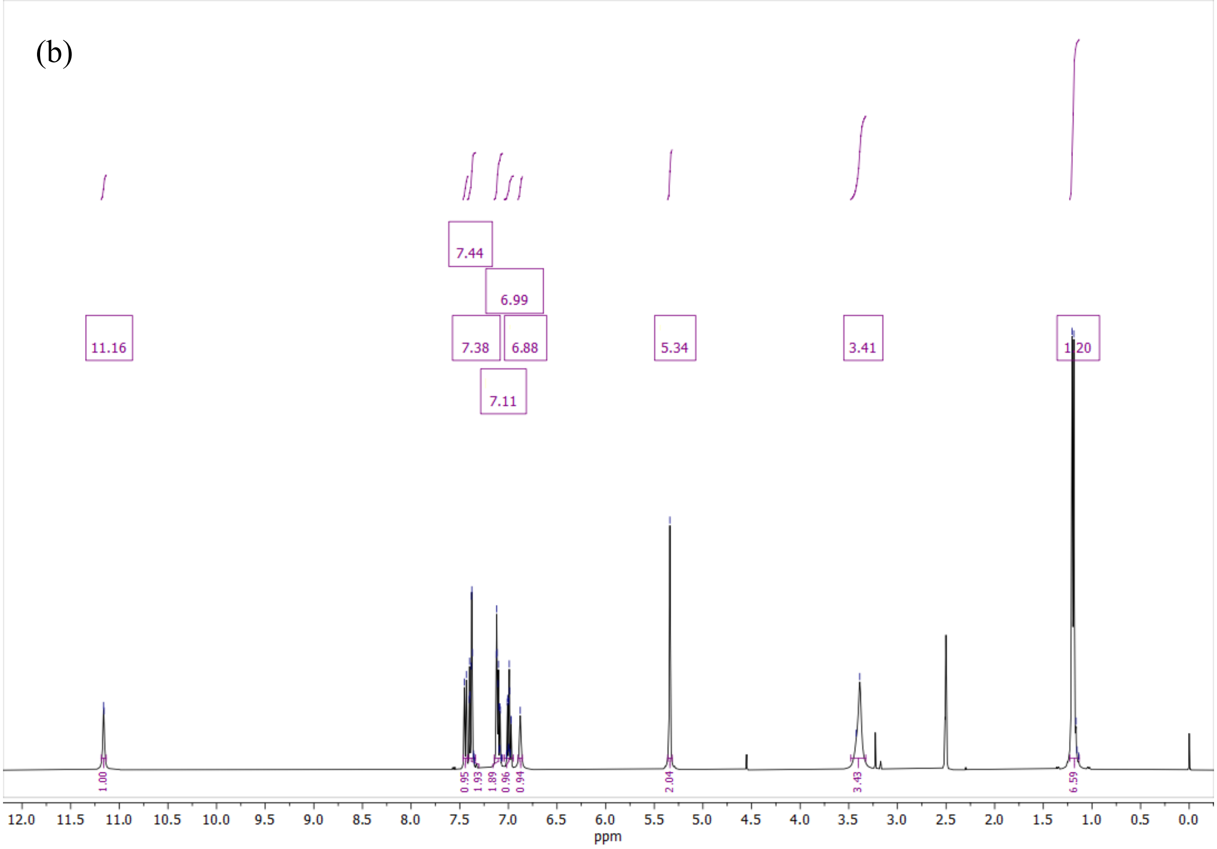

Figure S7 (a) ^{13}C NMR spectrum of complex **3** **(b)** ^{1}H NMR spectrum of complex **3**

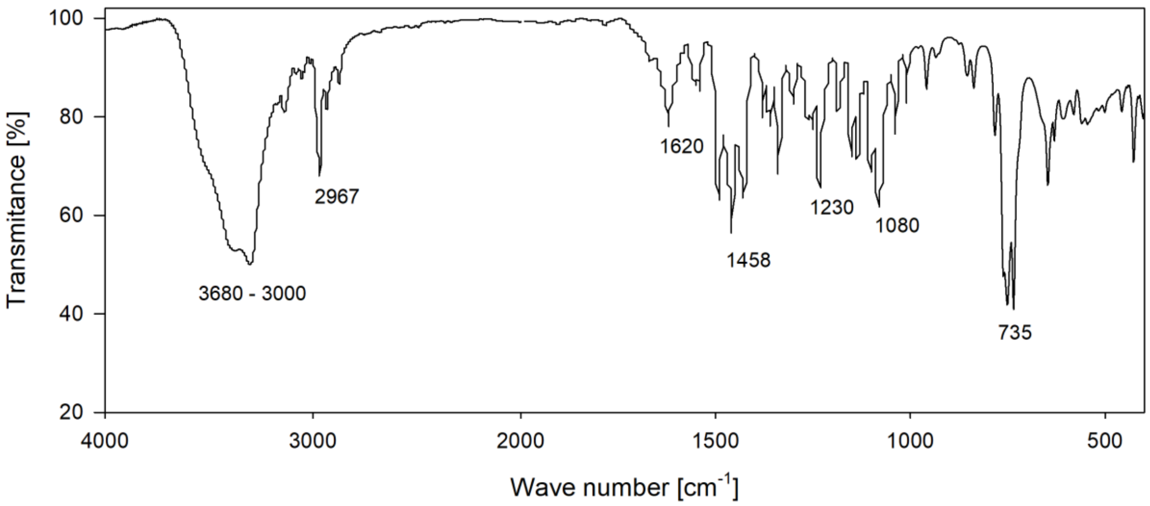

Figure S8. IR spectrum of complex **3**

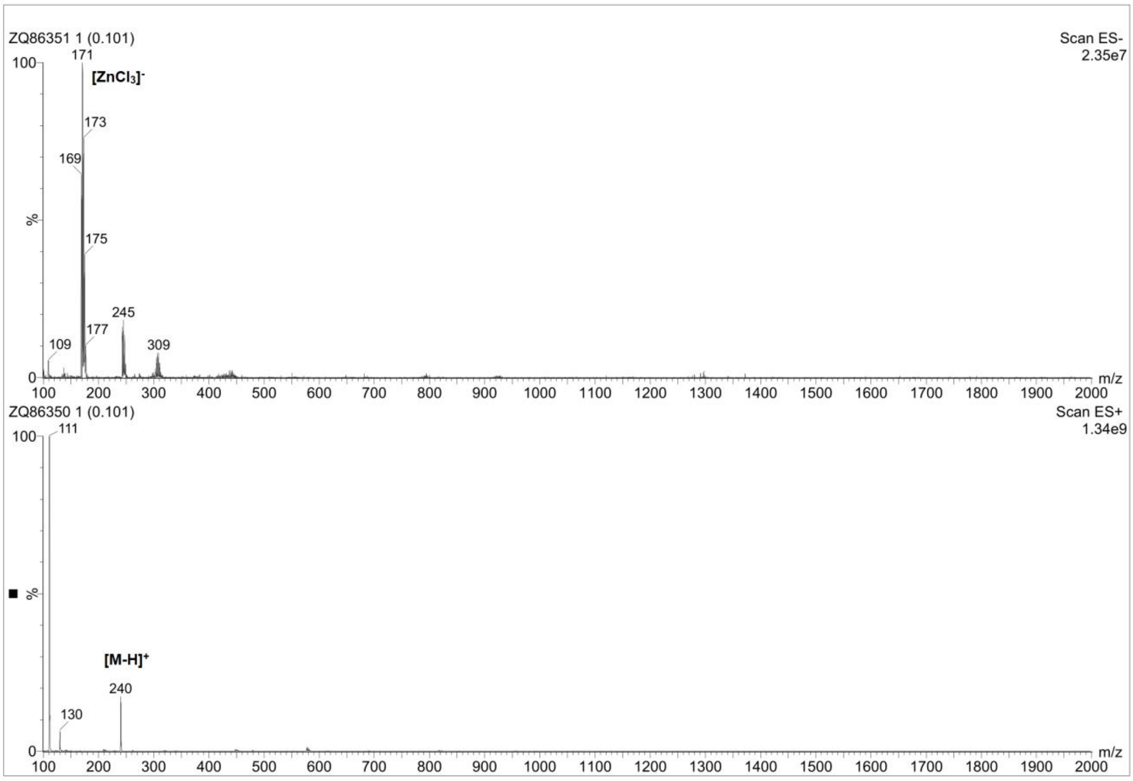

Figure S9. ESI-MS spectrum of complex **3**

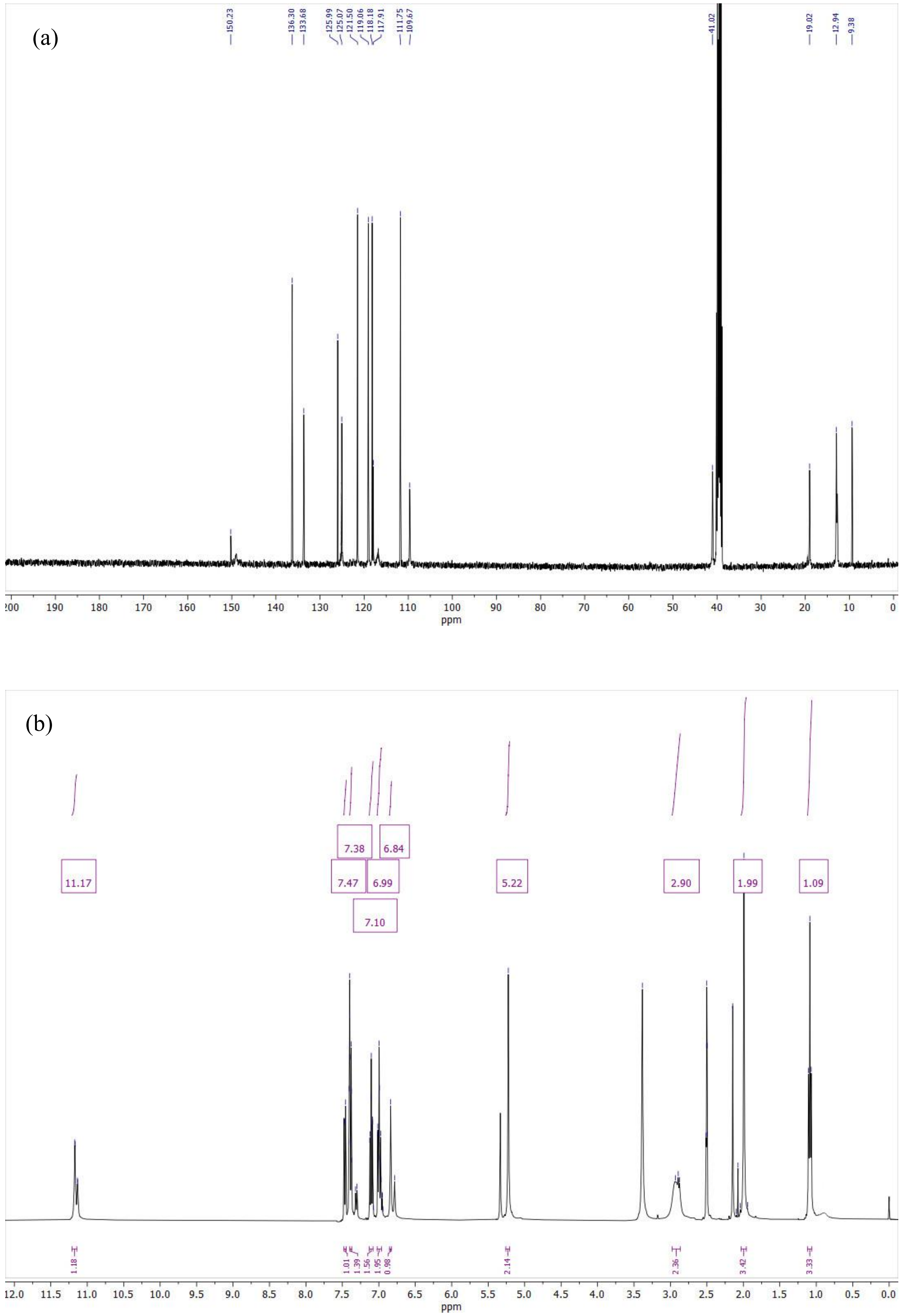

Figure S10 (a) ^{13}C NMR spectrum of complex **4** **(b)** ^{1}H NMR spectrum of complex **4**

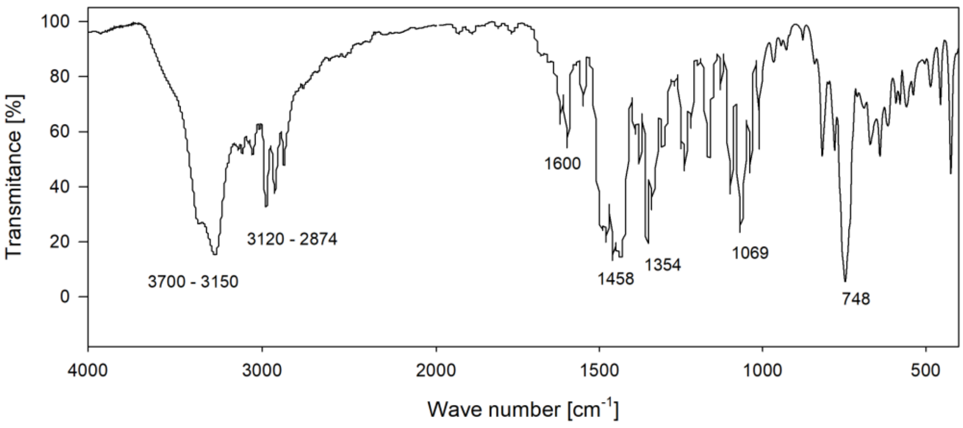

Figure S11. IR spectrum of complex **4**

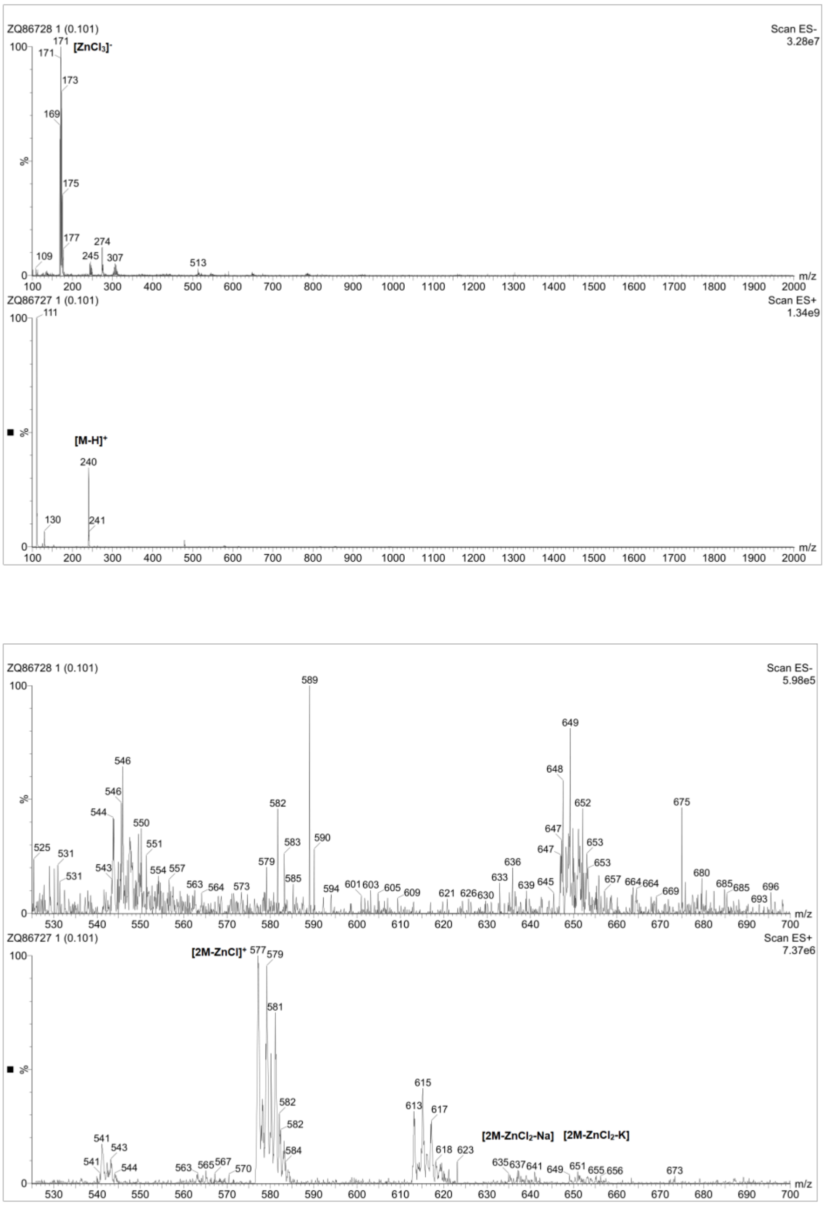

Figure S12. ESI-MS spectra of complex **4**

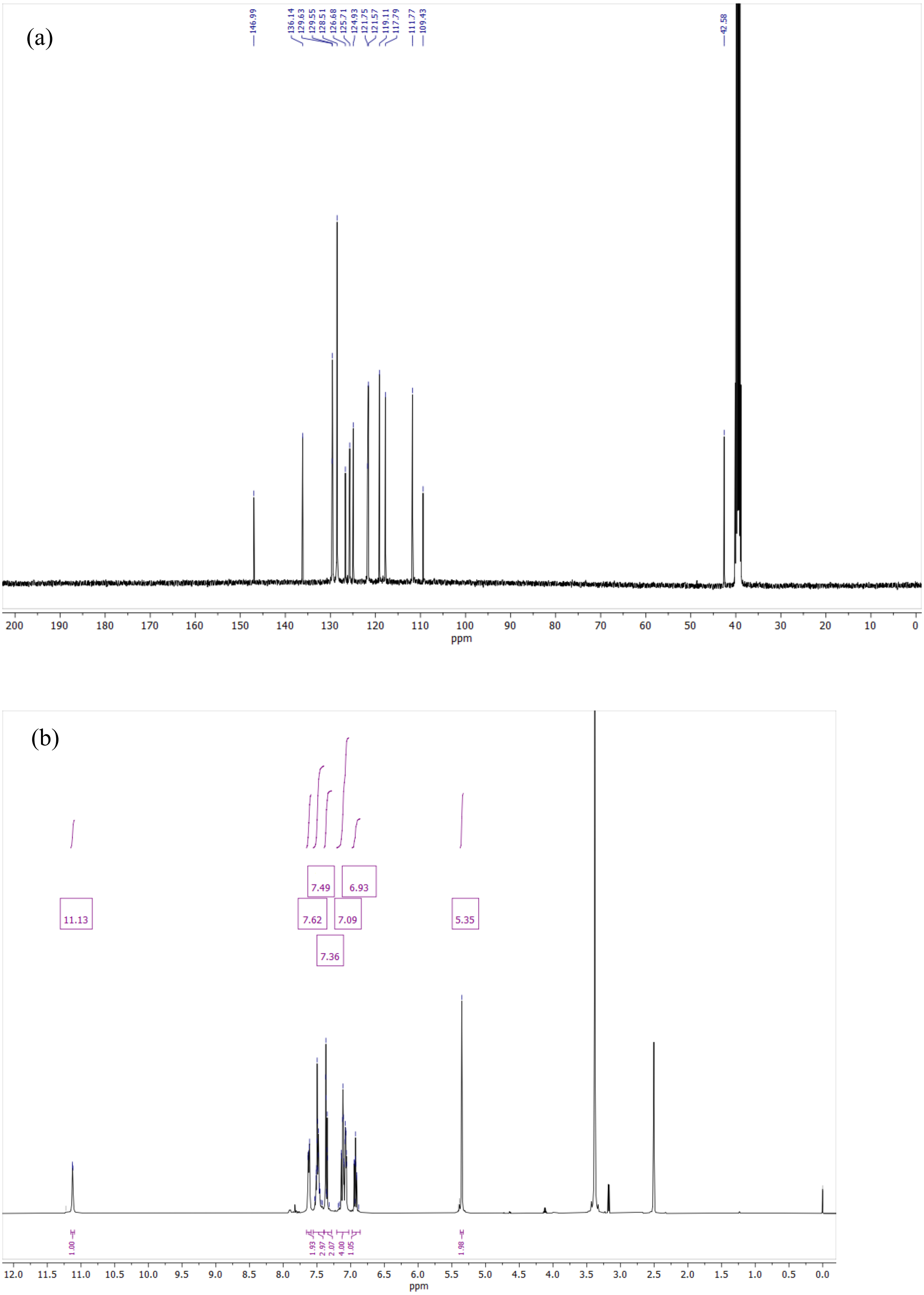

Figure S13 (a) ^{13}C NMR spectrum of complex **5** **(b)** ^{1}H NMR spectrum of complex **5**

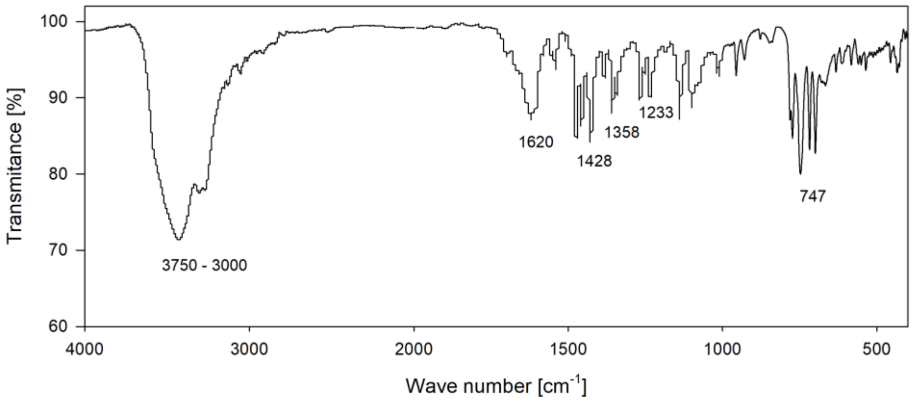

Figure S14. IR spectrum of complex **5**

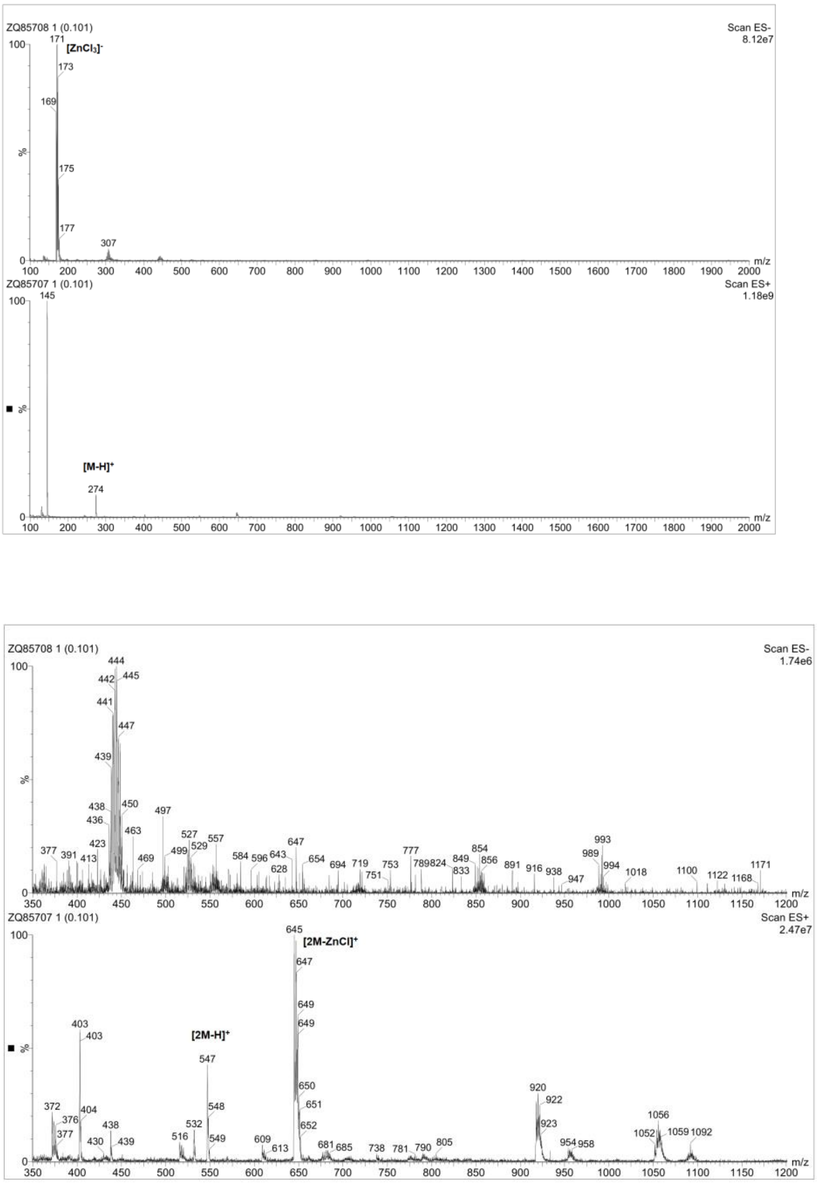

Figure S15. ESI-MS spectra of complex **5**

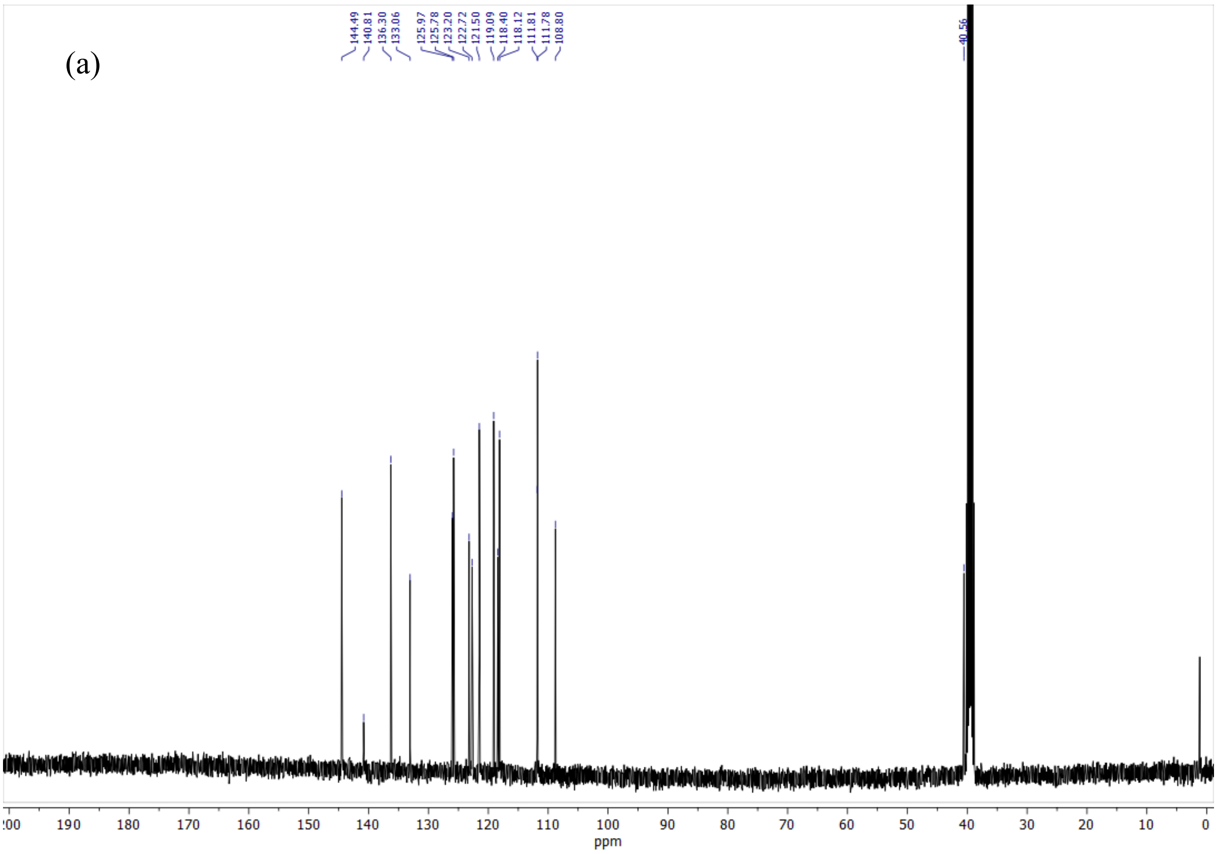

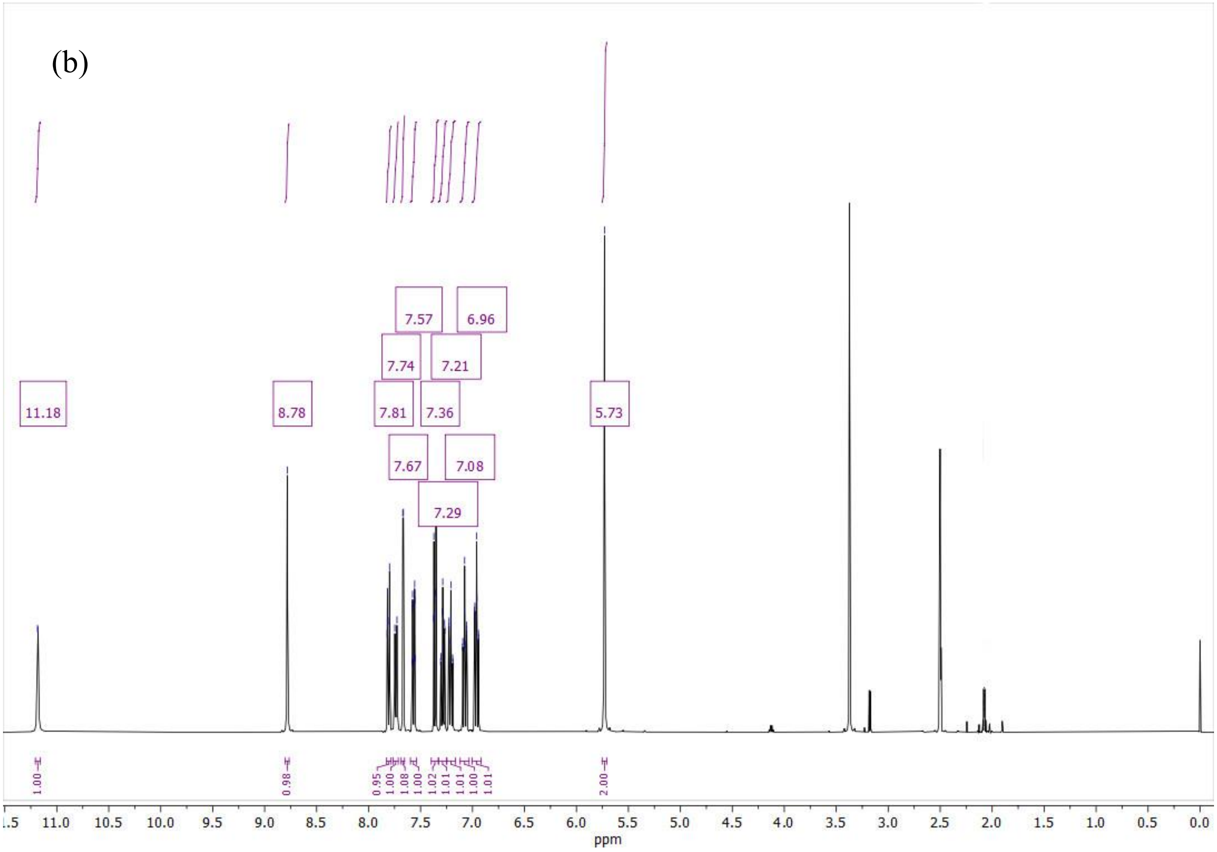

Figure S16 (a) ^{13}C NMR spectrum of complex **6** **(b)** ^{1}H NMR spectrum of complex **6**

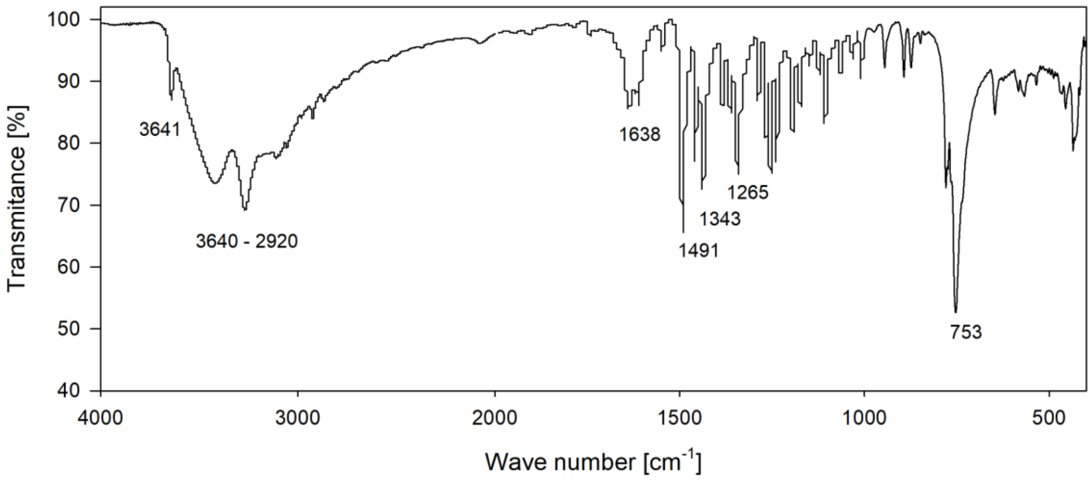

Figure S17. IR spectrum of complex **6**

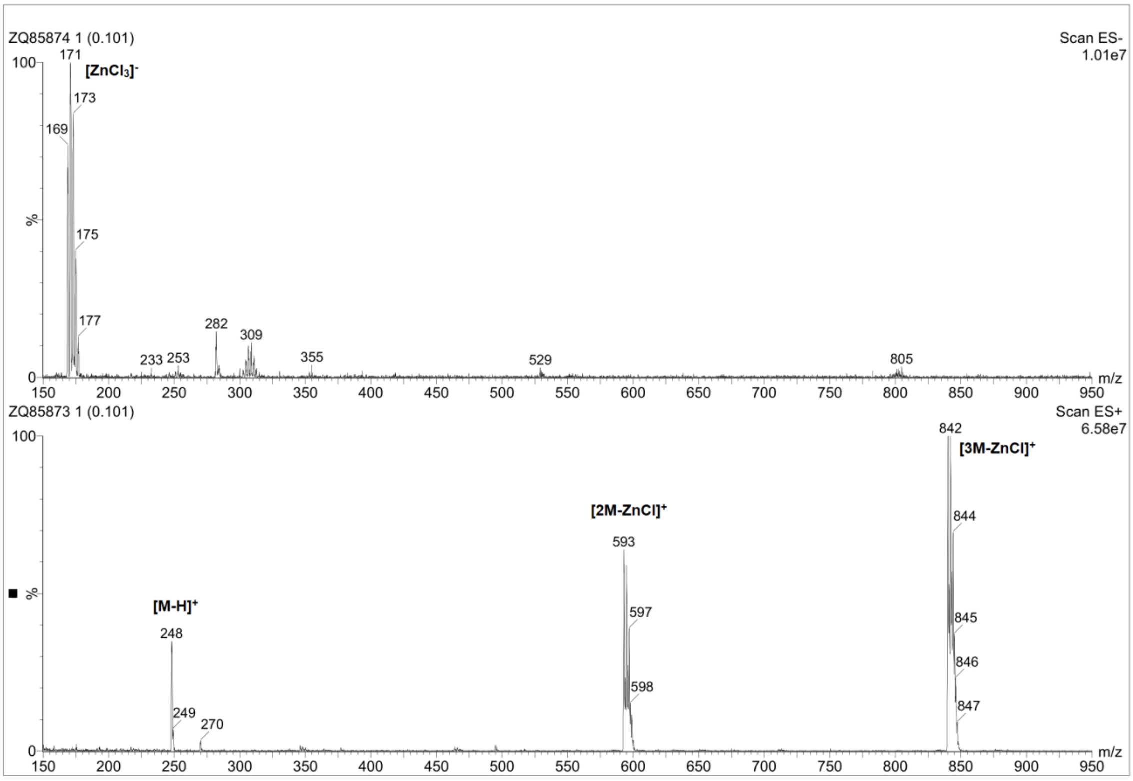

Figure S18. ESI-MS spectrum of complex **6**

Table S1. Selected bond lengths (Å) and valence angles (°) for complexes **1-5.**

	**1**	**2**	**3**	**4**	**5**
Zn1—N3<br>Zn1—N23	2.007 (3)	2.0140 (19)	2.020 (3)<br>2.020 (3)	2.022 (4)	2.000 (2)<br>2.005 (3)
Zn1—Cl1<br>Zn1—Cl2	2.2458 (9)	2.2607 (7)	2.2257 (14)<br>2.2348 (14)	2.2594 (15)	2.2543 (12)<br>2.2508 (12)
N3—Zn1—N3^{i}(N23)	105.83 (14)	110.53 (11)	102.68 (12)	108.9 (2)	112.56 (11)
N3—Zn1—Cl1<br>N23—Zn1—Cl2	111.16 (8)	112.31 (6)	110.05 (10)<br>110.27 (10)	106.37(11)	109.38 (9)<br>110.49 (9)
N3—Zn1—Cl1^{i}(Cl2)<br>N23—Zn1—Cl2	109.38 (8)	107.41 (6)	108.97 (11)<br>109.01 (11)	114.62(12)	109.69 (9)<br>106.88 (10)
Cl1—Zn1—Cl^{i}(Cl2)	109.89 (5)	106.87 (4)	115.13 (6)	106.19 (10)	107.68 (5)

Symmetry code(s): (i) -*x*+1, -*y*+1, *z* (for complexes **1** and **2**); -*x*+1, *y*, -*z*+3/2 (for complex 4).
